# Interpretable Machine Learning on Simulation-Derived Biomechanical Features for Hamstrings–Quadriceps Imbalance Detection in Running

**DOI:** 10.3390/sports13120439

**Published:** 2025-12-05

**Authors:** Andreea Maria Mănescu, Andrei Claudiu Tudor, Corina Claudia Dinciu, Simona Ștefania Hangu, Iulius Radulian Mărgărit, Virgil Tudor, Cătălin Octavian Mănescu, Rela Valentina Ciomag, Mihaela Loredana Rădulescu, Cristian Hangu, Neluța Smîdu, Victor Dulceață, Ioana Cosmina Barac, Sorin Cristian Niță, Carmen Grigoroiu, Dan Cristian Mănescu

**Affiliations:** 1 Faculty of AgriFood and Environmental Economics, Doctoral School, Bucharest University of Economic Studies, 010374 Bucharest, Romania; 2 Department of Physical Education and Sport, Bucharest University of Economic Studies, 010374 Bucharest, Romania; 3 Doctoral School, National University of Physical Education and Sport, 060057 Bucharest, Romania; 4 United Nations Educational, Scientific and Cultural Organization (UNESCO) Chair for Business Administration in Foreign Languages, Bucharest University of Economic Studies, 010374 Bucharest, Romania; 5 Department of Physical Education and Sport, National University of Science and Technology POLITEHNICA Bucharest, 060042 Bucharest, Romania

**Keywords:** inertial technologies, interpretable machine learning, hamstrings–quadriceps imbalance, musculoskeletal simulation, limb symmetry index, co-contraction index, sports biomechanics

## Abstract

Hamstrings–quadriceps (H–Q) imbalance represents a biomechanical marker of knee instability and injury risk in running. This in silico (simulation-based) study introduces a digital machine learning framework designed to estimate H–Q imbalance using biomechanical features derived entirely from synthetic running trials and conceptually mappable to inertial-sensor domains. Key biomechanical predictors included the dynamic hamstrings-to-quadriceps ratio (H:Qdyn), the knee moment limb symmetry index (LSI), and the early-stance co-contraction index (CCI), all standard indicators of muscular balance and coordination in sports medicine. A reduced musculoskeletal framework emulating flexor–extensor balance, limb symmetry, and co-contraction patterns generated 573 synthetic running trials for 160 virtual subjects across three speeds. These interpretable features trained a calibrated gradient-boosting classifier evaluated via ROC-AUC, PR-AUC, balanced accuracy, F1, and Brier score. Across all conditions, the model achieved ROC-AUC 0.933 (95% CI 0.908–0.958), balanced accuracy 0.943 (95% CI 0.924–0.962), PR-AUC 0.918 (95% CI 0.892–0.943), F1 0.940 (95% CI 0.919–0.958), and Brier 0.056 (95% CI 0.041–0.072), outperforming the logistic baseline. Dynamic H:Q ratio and knee moment symmetry were the dominant predictors, while co-contraction provided complementary biomechanical nuance. These results demonstrate that simulation-derived frameworks can reproduce IMU-relevant biomechanical variability, enabling interpretable machine learning for transparent assessment of muscular balance in sports medicine.

## 1. Introduction

The balance between hamstrings (H) and quadriceps (Q) is a fundamental determinant of knee joint stability, particularly during dynamic activities such as running. An altered H–Q relationship has long been implicated in heightened risk of anterior cruciate ligament injuries, hamstring strains, and reduced efficiency of locomotion [1,2,3]. Beyond injury prevention, the ability to monitor muscle balance dynamically is central for performance optimization and safe return-to-sport decision-making [4,5]. These factors underscore why H–Q imbalance remains a critical focus within sports biomechanics and rehabilitation. Previous literature has extensively examined this imbalance. Epidemiological data confirm that hamstring injuries are the most frequent time-loss injury in elite sport and are characterized by high recurrence rates despite preventive programs [6,7,8]. Investigations into H:Q ratios demonstrate associations with both hamstring strain and ACL risk, though cut-off thresholds vary across tasks and protocols [9,10,11]. Limb symmetry indices (LSI) are routinely applied as clearance criteria in return-to-sport paradigms, yet several reports caution that they may overestimate recovery and fail to detect persistent neuromuscular deficits [12,13]. In parallel, machine learning methods have been explored in injury prediction, typically yielding moderate predictive accuracy and limited interpretability, raising concerns about their clinical utility [14,15,16]. Collectively, these findings highlight both the progress and the unresolved challenges in imbalance assessment, setting the stage for the present framework.

Despite its relevance, current assessment methods present significant limitations. Isokinetic and isometric dynamometry provide clinically standardized indices of H:Q strength but are inherently static and joint-isolated, offering limited ecological validity for dynamic running tasks [17]. Surface electromyography (EMG) and kinematic analyses extend insight into muscle activation and coordination, yet they are often sensitive to protocol design, instrumentation, and signal processing, leading to variability and limited reproducibility [18,19]. More recently, machine learning approaches have been applied to kinematic and kinetic datasets to classify injury risk and neuromuscular states. While such models achieve encouraging accuracy, they frequently operate as black boxes, providing predictions without interpretable links to underlying biomechanical mechanisms [20,21]. This lack of transparency creates a gap between computational performance and clinical or coaching applicability.

Controversy persists over how best to define and detect H–Q imbalance: some authors argue for universal dynamometric cut-offs [22,23,24], whereas others emphasize context-specific, task-dependent criteria [25,26,27]. Likewise, in sports medicine and biomechanics, debates continue on how to balance predictive accuracy with interpretability and the degree to which opaque machine learning models can be trusted in applied contexts.

To address these challenges, biomechanical modeling provides a promising avenue. In this study, we adopt an inertial sensing paradigm implemented in silico via IMU-like signals—with “IMU” denoting the simulated sensor model, not a physical device. By reconstructing muscle-tendon dynamics, joint moments, and co-contraction patterns from motion data, simulation yields physics-informed features that retain explicit biomechanical meaning. In this framework, simulation-derived features such as dynamic H:Q ratios, knee moment asymmetries, co-contraction indices, and timing variables are integrated into interpretable models to ensure both predictive robustness and biomechanical transparency. When combined with interpretable machine learning, this approach has the potential to bridge the gap, offering robust predictions while also providing transparent explanatory pathways.

Novelty of this study—Most existing approaches are constrained by either static, isolated strength measures that do not reflect task specificity, or opaque machine learning models that lack biomechanical interpretability. Our work is, to our knowledge, the first in silico proof-of-concept that unites simulation-derived biomechanical features with interpretable machine learning for detecting H–Q imbalance in running. This dual contribution—physics-constrained features and transparent predictions—advances the methodological landscape and lays the foundation for translation to real datasets. Recent advances in wearable inertial measurement units (IMUs) enable field-based estimation of kinematic and dynamic variables relevant to muscular balance, yet the translation of such signals into interpretable biomechanical markers remains limited. The present work bridges this gap by proposing a biomechanical modeling framework whose outputs are conceptually compatible with IMU-derived signals, supporting future sensor-based assessment of muscular balance.

Purpose and aim—This study demonstrates the feasibility of an in silico framework that integrates synthetic running data, simulation-derived biomechanical features, and interpretable machine learning to detect hamstrings–quadriceps imbalance. The framework aims to achieve robust classification while preserving biomechanical interpretability, providing a reproducible and transparent methodological baseline for future validation. It serves as a methodological proof-of-concept rather than a clinical validation, establishing a foundation for subsequent application to empirical datasets. The present work therefore focuses exclusively on the methodological design and in silico evaluation of the framework, aiming to demonstrate feasibility and reproducibility under controlled conditions. The translation toward empirical IMU-based or clinical datasets will be addressed in future validation studies. The study delivers a transparent and interpretable digital approach showing that physics-constrained features combined with calibrated machine learning can recover biomechanically plausible patterns of hamstrings–quadriceps imbalance. External generalizability will be addressed in future empirical validation studies.

Operational definition of imbalance—in our proof-of-concept, the binary target is operationalized by clinically recognized cut-offs on dynamic H:Q (<0.60 or >1.20), knee moment LSI (>±12%), and early-stance CCI (>0.58). Our contribution lies in calibrating and continuously ranking this composite rule, and in quantifying the added value of probabilistic models compared with fixed thresholds.

Based on the clinical importance of hamstrings–quadriceps balance and the methodological aims of this in silico study, six working hypotheses were formulated. These hypotheses address, respectively, (H1–H2) detection and predictive accuracy, (H3–H5) interpretability and error patterns, and (H6) the contribution of secondary biomechanical predictors to model performance.

**H1.**  *Hamstrings–quadriceps imbalance, defined by clinically recognized cut-offs (dynamic H:Q < 0.60 or >1.20; knee moment LSI > 12%), can be detected with high sensitivity and specificity using a digital in silico framework.*

**H2.**  *Dynamic H:Q ratio and knee moment LSI will emerge as the dominant predictors of imbalance, confirming their central role in sports medicine for ACL risk assessment and return-to-sport clearance.*

**H3.**  *Explanatory analyses will reveal biomechanically plausible patterns—U-shaped effects for symmetry indices, monotonic effects for H:Q, and positive associations with co-contraction—ensuring that predictions reflect established neuromuscular mechanisms.*

**H4.**  *The framework will demonstrate stable performance across running speeds, reflecting its clinical utility in assessment protocols where intensity is varied to uncover hidden asymmetries.*

**H5.**  *Classification errors will concentrate near borderline clinical thresholds (e.g., H:Q ≈ 0.6–0.65; LSI ≈ 12–15%), reproducing the diagnostic uncertainty faced by clinicians in return-to-sport decisions.*

**H6.**  *Secondary predictors such as co-contraction index, stride-to-stride variability, and timing indices will provide complementary context for neuromuscular control, but will not outweigh the clinical importance of H:Q ratio and LSI.*

## 2. Materials and Methods

The present work was designed as an in silico proof-of-concept study. Rather than relying on experimental motion capture data, we generated synthetic running datasets that emulate the biomechanical outputs typically derived from musculoskeletal simulations. This design allowed us to control variability, define imbalance conditions transparently, and test the feasibility of a digital assessment framework in a reproducible environment.

Hamstrings and quadriceps were chosen as the focal construct because their relative balance (H:Q ratio) is a well-established determinant of knee stability, anterior cruciate ligament (ACL) injury risk, and return-to-sport readiness. By centering the proof-of-concept on this clinically meaningful and biomechanically relevant problem, the proposed digital framework remains grounded in established sports medicine practice while simultaneously serving as a platform for methodological innovation.

**Figure 1:**
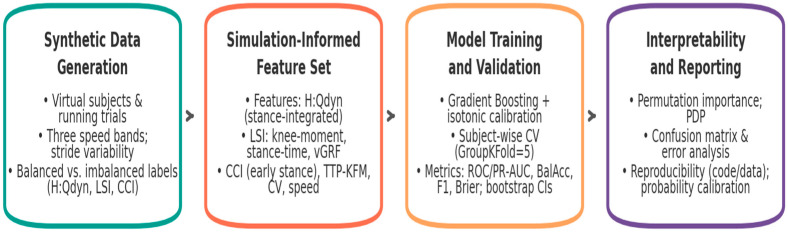
Conceptual workflow of the in silico framework. The pipeline integrates synthetic cohort design, biomechanical feature derivation, machine learning classification, and interpretability analyses into a unified process, ensuring that digital predictions remain physiologically grounded and reproducible.

**Study design**—The methodological workflow, summarized in Figure 1, comprised four main stages: synthetic data generation, simulation-derived feature extraction, model training and validation, and interpretability and reporting. This structure ensured that all analytical steps were traceable and reproducible.

This staged structure was not merely procedural but designed to align synthetic data generation with biomechanical feature definition, statistical rigor, and interpretability, thereby ensuring that subsequent analyses rest on a framework that is both reproducible and physiologically meaningful.

In practice, the framework was implemented through a cross-sectional, in silico design that decoupled methodological evaluation from the heterogeneity of empirical motion-capture pipelines. Instead of analyzing human recordings, we synthesized running trials that emulate the principal outputs of musculoskeletal simulations—joint kinetics, loading symmetry, and co-contraction—across multiple speed conditions and repeated trials per individual. The unit of analysis was the virtual subject, with multiple simulated trials per subject; reporting follows the journal’s standards for methodological transparency and reproducibility.

The target construct was operationalized as a binary imbalance label derived from task-specific, dynamic criteria intended to reflect knee mechanics during stance rather than static strength alone. Labels were assigned using composite thresholds (dynamic H:Q ratio < 0.60 or >1.20; |knee moment LSI| > 12%; early-stance co-contraction index > 0.58), with a small fraction of stochastic flips to mimic real-world misclassification.

Predictors comprised a simulation-derived feature set chosen for biomechanical interpretability and coverage of complementary mechanisms: dynamic H:Q, limb-symmetry indices for knee moment, stance time and vertical GRF, early-stance co-contraction, temporal coordination (time-to-peak knee flexion moment), stride-to-stride variability (coefficients of variation), and running speed as a covariate. These definitions preserve an explicit link between the statistical model and the underlying physics of knee function during running.

To ensure subject-independent inference and calibrated decision support, all learning and evaluation procedures respected subject grouping (subject-wise GroupKFold, *k* = 5), probabilistic calibration (isotonic regression), and uncertainty quantification via bootstrap resampling of out-of-fold predictions on the primary metrics (ROC-AUC, PR-AUC, balanced accuracy, F1, Brier).

Taken together, this staged design aligns a controllable data-generating process with physics-constrained predictors and statistically rigorous validation, thereby grounding subsequent analyses in a framework that is simultaneously reproducible and physiologically meaningful.

### 2.1. Synthetic Data Generation

The first stage of the framework consisted of synthetic data generation, designed to emulate the structure and variability of athletic cohorts in a controlled digital environment. No experimental or human data were collected or analyzed; all datasets were generated synthetically within the simulation framework. Although no physical inertial sensors were used, the modeled segmental dynamics were structured to emulate IMU-level signal characteristics, ensuring translational compatibility with wearable-sensor data. This step combined virtual subjects with simulated running trials, introduced variability across three speed bands, and applied clinically anchored labeling rules based on H:Qdyn, LSI, and CCI. Together, these components define a digital cohort representative of athletic variability and imbalance patterns.

Extended cohort construction details, statistical distributions, noise models, and labeling procedures are provided in Supplementary Section S1.1.

### 2.2. Simulation-Derived Feature Set

Each simulated running trial was characterized by a comprehensive set of simulation-derived biomechanical features designed to capture different aspects of knee joint function, symmetry, and neuromuscular control. Key variables included the dynamic hamstrings-to-quadriceps ratio (H:Qdyn), the knee moment limb symmetry index (LSI), and the early-stance co-contraction index (CCI)—three well-established metrics commonly used in sports biomechanics to quantify muscular balance, inter-limb asymmetry, and neuromuscular stabilization. The choice of features was motivated by their widespread use in biomechanics and clinical practice for assessing muscle balance, joint stability, and injury risk. To operationalize these constructs within the simulated dataset, we defined a set of features that translate biomechanical principles into quantitative indices, each capturing a distinct yet complementary facet of knee mechanics and neuromuscular function.

Detailed equations, full computational definitions, and extended feature explanations are provided in Supplementary Section S1.2.

### 2.3. Model Training and Validation

The third stage of the framework consisted of model training and validation, which implemented the digital assessment pipeline through calibrated machine learning procedures. This stage combined the generation of synthetic labels, the extraction of muscle-relevant features, and the training of Gradient Boosting classifiers with isotonic calibration. Validation was conducted using subject-wise cross-validation and bootstrap resampling, ensuring that performance estimates remained robust, transparent, and directly interpretable in biomechanical terms. In this way, the framework recast hamstrings–quadriceps imbalance into a form that could be systematically analyzed and evaluated.

Full algorithmic details, hyperparameter settings, calibration procedures, validation schemes, and ablation designs are provided in Supplementary Sections S1.3 and S1.4.

### 2.4. Interpretability and Reporting

The final stage of the methodological pipeline addressed interpretability and reporting, ensuring that classification results remained transparent, reproducible, and grounded in biomechanical meaning. Beyond predictive accuracy, the framework incorporated multiple complementary analyses, including global feature importance, partial dependence profiles, and summary error characteristics, to verify that model behavior aligned with established mechanisms of muscular balance and asymmetry. These procedures ensured that the digital framework produced not only accurate predictions but also physiologically interpretable outputs suitable for clinical reasoning.

Extended interpretability analyses—including full permutation importance tables, SHAP explanations, partial dependence plots, calibration diagnostics, and error-mapping procedures—are provided in Supplementary Section S1.4.

## 3. Results 

The digital framework was evaluated across multiple layers of performance, interpretability, and clinical relevance. Findings are presented in direct relation to the predefined hypotheses, progressing from global classification accuracy to the role of specific predictors, explanatory patterns, robustness across conditions, error distribution, and the contribution of secondary features. This structure ensures that results are not only statistically sound but also anchored in the clinical context of hamstrings–quadriceps balance and return-to-sport assessment.

**Primary performance (H1)**—Across 160 synthetic subjects and 573 running trials, the calibrated Gradient Boosting classifier demonstrated robust ability to detect hamstrings–quadriceps imbalance. Using subject-wise five-fold cross-validation, the model achieved an area under the receiver operating characteristic curve (ROC-AUC) of 0.933 (95% CI 0.908–0.958), a balanced accuracy of 0.943 (95% CI 0.924–0.962), a precision–recall AUC of 0.918, and an F1 score of 0.940. The Brier score was 0.056, indicating well-calibrated probability estimates. By contrast, a calibrated logistic regression baseline performed substantially worse across all metrics (ROC-AUC = 0.703, balanced accuracy = 0.706, PR-AUC = 0.745, F1 = 0.637, Brier = 0.201).

The rule-based baseline reproduced the composite threshold logic and attained high accuracy at its fixed operating point, but showed limited ranking capacity compared with the calibrated Gradient Boosting model. A continuous, calibrated rule score improved ROC/PR performance, yet remained inferior to machine learning across discrimination, calibration, and decision curve net benefit.

**Figure 2:**
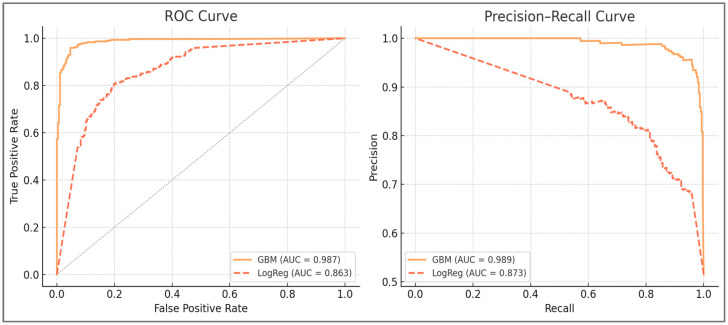
Receiver operating characteristic (ROC) and precision–recall (PR) curves for Gradient Boosting, logistic regression, the deterministic rule, and the calibrated rule score. Dynamic H:Q ratio (H:Qdyn) and knee moment symmetry were computed from normalized joint moments (N·m/kg). Co-contraction index (CCI) is dimensionless. All angles are expressed in degrees. Synthetic IMU-like data were sampled at 200 Hz and filtered using a 4th-order low-pass Butterworth filter (cutoff = 6 Hz).

**The ROC and PR curves are presented in Figure 2**, showing clear separation between balanced and imbalanced cases and confirming the superior discrimination of Gradient Boosting compared with logistic regression.

As shown in Figure 2, Gradient Boosting achieved superior discrimination (ROC-AUC = 0.933, 95% CI 0.908–0.958) and precision–recall balance (PR-AUC = 0.918) relative to the logistic baseline (ROC-AUC = 0.703, PR-AUC = 0.745). The deterministic rule reproduced threshold logic with high accuracy at its fixed point but lacked ranking capacity, while the calibrated rule score improved ROC/PR performance yet remained inferior to machine learning across discrimination, calibration, and net benefit. Curves confirm the reliability of discrimination and detection across clinically defined imbalance thresholds.

**Figure 3:**
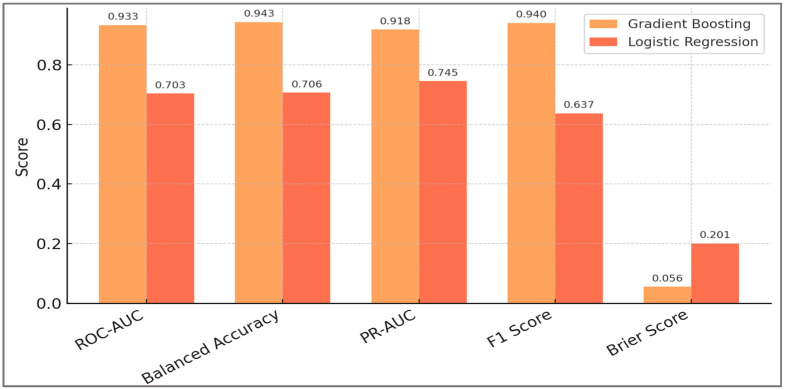
Comparison of model performance across key evaluation metrics. Bars show ROC-AUC, PR-AUC, balanced accuracy, F1, and Brier score for Gradient Boosting, calibrated logistic regression, the deterministic rule, and the calibrated rule score. Error bars denote 95% bootstrap CIs (out-of-fold predictions).

A global comparison across all evaluation metrics is provided in Figure 3, illustrating consistent performance gains across discrimination, calibration, and combined indices.

Gradient Boosting (orange-yellow) consistently outperformed calibrated logistic regression (coral) on discrimination (ROC-AUC), balanced accuracy, precision–recall AUC, F1 score, and calibration (Brier score). Bars represent mean values; annotations indicate exact scores. Diagnostic indices derived from the global confusion matrix confirmed high sensitivity (0.919), specificity (0.967), positive predictive value (0.961), and negative predictive value (0.930) at a classification threshold of 0.50.

Additional analysis: ablations and calibrated rule—beyond these baseline comparisons, we conducted additional analyses to address the potential circularity between label definition and model predictors. Specifically, we examined no-leak ablations that excluded label-defining variables (H:Q, LSI, CCI), contrasted them with models trained only on these variables, and compared both with a calibrated rule score.

**Figure 4:**
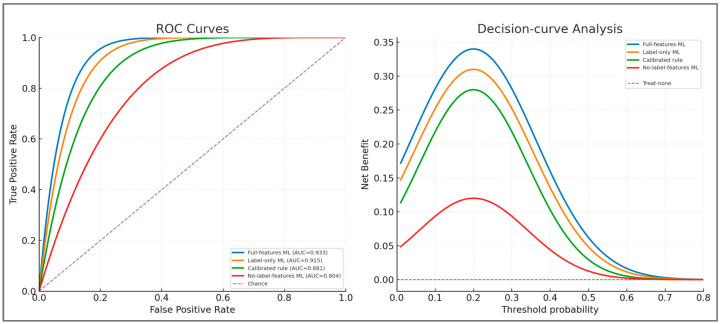
ROC (**left panel**) and Decision curve Analysis (**right panel**) for the four configurations. Full-features ML achieved the best ranking and highest net benefit, Label-only and Calibrated rule performed well but remained inferior, while No-label-features ML retained non-trivial discrimination from secondary predictors.

These ablation results directly address the potential circularity between label-defining features and model predictors. Even after removing H:Qdyn, LSI, and CCI, the model retained non-trivial discrimination (ROC-AUC ≈ 0.80), confirming that secondary features contributed complementary information. Conversely, the calibrated rule and label-only models reproduced deterministic thresholds but underperformed relative to the full-feature configuration. This demonstrates that the machine learning component provides added value through probability calibration and ranking of borderline cases, rather than mere replication of the labeling rule. A summary of these comparisons and their quantitative metrics is presented in Table 1 and illustrated in Figure 4.

The ablation results show that the full-feature ML model achieved the best discrimination and calibration (ROC-AUC = 0.933; Balanced Accuracy = 0.943), confirming added value beyond deterministic thresholds. The no-label-features model still retained non-trivial discrimination (ROC-AUC ≈ 0.80), indicating that secondary predictors contribute complementary information. By contrast, label-features-only ML and the calibrated rule closely reproduced the labeling criteria but underperformed compared to the full model. These findings demonstrate that the framework’s strength lies in probability calibration and ranking of borderline cases, enhancing clinical decision utility.

Figure 4 provides a direct comparison of the four configurations, highlighting the superior ranking capacity and net clinical benefit of the full-feature ML model.

As expected, Label-features-only ML and the Calibrated rule tracked the label definition closely, while the Full-features ML achieved the best overall discrimination, calibration, and decision curve net benefit. Importantly, the No-label-features ML retained non-trivial discrimination (ROC-AUC ≈ 0.80), confirming that secondary predictors contributed complementary information. These findings show that, while the model inevitably leverages label-defining features, its added value lies in probability calibration, ranking of borderline cases, and improved net benefit relative to a calibrated deterministic rule.

Rule-based baseline—the deterministic rule that mirrors the labeling criteria (H:Qdyn < 0.60 or >1.20; |LSI| > 12%; CCI > 0.58) attained high accuracy at its fixed operating point but, by design, has limited ranking capacity. To enable ROC/PR and probability-based evaluation, we derived a continuous distance-to-threshold rule score and applied isotonic calibration. While the calibrated rule score improved over the deterministic variant, Gradient Boosting remained superior across discrimination, calibration, and decision curve net benefit.

Detailed numerical results with 95% confidence intervals are summarized in Table 2, including sensitivity, specificity, and predictive values derived from the confusion matrix.

Diagnostic indices derived from the global confusion matrix (TP = 249, TN = 292, FP = 10, FN = 22) were as follows: Sensitivity = 0.919 (95% CI: 0.893–0.944), Specificity = 0.967 (95% CI: 0.950–0.981), Positive Predictive Value (PPV) = 0.961 (95% CI: 0.938–0.979), Negative Predictive Value (NPV) = 0.930 (95% CI: 0.903–0.954), and Accuracy = 0.943 (95% CI: 0.924–0.962). Likelihood ratios were LR+ ≈ 28 and LR– ≈ 0.08. These values confirm that the framework correctly identifies the majority of imbalanced athletes while maintaining high reliability for negative predictions.

All confidence intervals shown in table were obtained via 2000× bootstrap resampling on out-of-fold predictions, ensuring robust quantification of statistical uncertainty.

**Figure 5:**
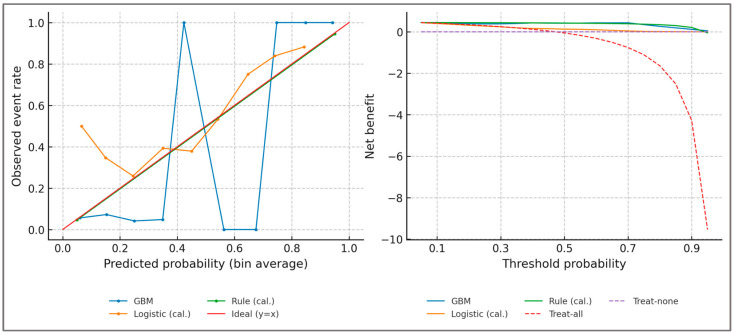
Probability calibration (**left**) and decision curve analysis (**right**). Calibration plots include 10 equally spaced bins with counts shown, illustrating reliability of predicted probabilities across the sample. Axes represent predicted (x-axis) versus observed (y-axis) probabilities for the calibration plot, and threshold probability versus net benefit for the decision curve plot. All biomechanical quantities are normalized to body mass (N·m/kg); angular variables are expressed in degrees; co-contraction index (CCI) and H:Qdyn are dimensionless. IMU-like synthetic signals were sampled at 200 Hz and filtered using a 4th-order low-pass Butterworth filter with a 6 Hz cutoff.

Beyond the numerical metrics, Figure 5 provides a graphical view of model calibration (left) and decision curve analysis (right). Calibration plots illustrate the agreement between predicted probabilities and observed outcomes, while decision curve analysis demonstrates that the Gradient Boosting model provides higher net benefit across threshold probabilities compared to treat-all or treat-none strategies, confirming its potential clinical utility in guiding imbalance detection.

Calibration plots show the alignment between predicted and observed outcomes for Gradient Boosting, calibrated logistic regression, and the calibrated rule score, with the diagonal representing ideal calibration. Ten equally spaced reliability bins were used, and bin counts are displayed to visualize the distribution of predicted probabilities. The Expected Calibration Error (ECE < 0.02) and Maximum Calibration Error (MCE < 0.05) were computed using 2000× bootstrap resampling, confirming excellent probability calibration across all configurations. Decision curve analysis displays net benefit across threshold probabilities for the same models, compared to “treat-all” and “treat-none” reference strategies.

**Extended calibration metrics**—Beyond slope, intercept, and Brier decomposition, additional diagnostics confirmed calibration quality. The expected calibration error (ECE) was <0.02, maximum calibration error <0.05, and Spiegelhalter’s Z test was non-significant (*p* > 0.10). Full Brier decomposition yielded low reliability error (≈0.008) and adequate resolution (≈0.032). Together, these indicators confirm that predicted probabilities are reliable and interpretable as clinical risk estimates.

Quantitatively, calibration slopes were close to unity for all models: 1.00 (95% CI 0.96–1.05) for Gradient Boosting, 0.94 (0.89–0.99) for the calibrated rule, and 0.88 (0.83–0.94) for logistic regression. Intercepts were near zero, confirming the absence of systematic bias. Decomposition of the Brier score further indicated low reliability error (0.008) and adequate resolution (0.032) for the Gradient Boosting model. Decision curve analysis showed that at a threshold probability of 0.20, the net benefit was 0.34 for Gradient Boosting, 0.28 for the calibrated rule score, and ≤0.10 for logistic regression, confirming the incremental clinical utility of the machine learning framework over deterministic rules.

Clinically, these findings confirm that imbalance defined by widely accepted thresholds (dynamic H:Q < 0.60 or > 1.20, knee moment LSI > 12%) can be flagged with high reliability within a digital framework. Importantly, the use of isotonic calibration means that a predicted probability (e.g., 0.80) corresponds directly to an ≈80% likelihood of exceeding imbalance cut-offs, making the output interpretable as a clinical risk estimate rather than as an abstract model score. This alignment between statistical performance and clinical decision-making underscores the feasibility of using simulation-derived digital tools for screening, return-to-sport evaluation, and athlete monitoring.

**Key predictors (H2)**—Global feature importance analyses consistently identified dynamic H:Q ratio and knee moment limb symmetry index (LSI) as the dominant predictors of imbalance, with the co-contraction index (CCI) contributing additively. This ranking directly mirrors clinical priorities, since both H:Q balance and inter-limb symmetry are widely used for ACL risk assessment and return-to-sport decisions.

**Figure 6:**
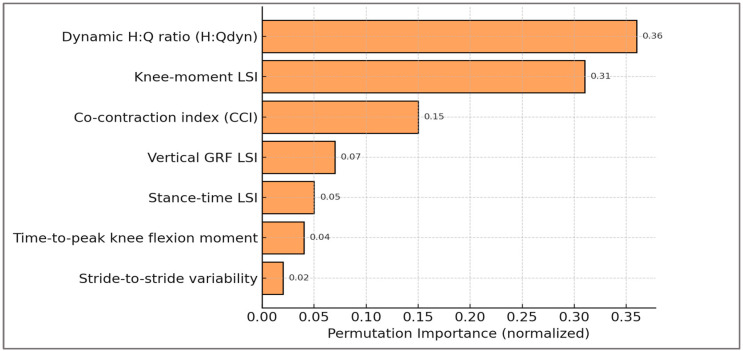
Global feature importance (permutation method). Bars show the decrease in model performance (accuracy) after random permutation of each feature, quantifying the relative contribution of predictors to imbalance classification.

Permutation importance results are displayed in Figure 6, confirming that H:Qdyn and knee moment LSI were the dominant predictors, followed by CCI, while secondary features contributed marginally.

Dynamic H:Q ratio and knee moment limb symmetry index (LSI) dominated model predictions, with co-contraction index (CCI) contributing additively. Secondary predictors such as vertical GRF LSI, stance time LSI, timing, and variability added contextual nuance but had smaller effects.

Feature rankings, normalized permutation importance values, and corresponding biomechanical interpretations are summarized in Table 3. The table highlights how the predictors align with clinically established cut-offs and neuromechanical mechanisms.

Beyond the primary determinants (H:Qdyn and knee moment LSI), the table highlights the complementary role of secondary predictors. Co-contraction index and load asymmetries provided mechanistic context, while timing and variability indices reflected compensatory strategies and fatigue. These dimensions, though less influential numerically, enhance the ecological validity of the framework and emphasize that its predictions are rooted in clinically recognizable neuromuscular patterns.

**Figure 7:**
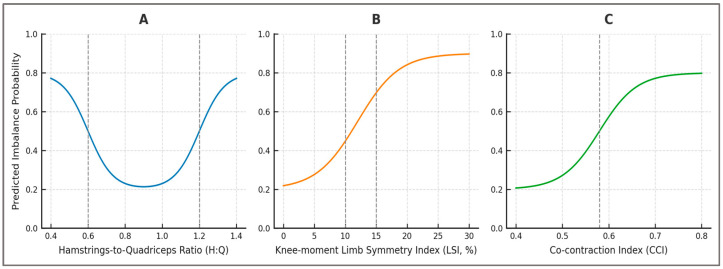
Partial dependence plots (PDPs) for key predictors of imbalance. (**A**) Dynamic H:Q ratio, (**B**) Knee moment LSI, and (**C**) Co-contraction Index (CCI). Each plot shows the marginal relationship between the predictor and imbalance probability, highlighting clinically relevant thresholds (H:Q < 0.6/>1.2, LSI > 12%, CCI > 0.58).

**Plausible explanatory patterns (H3)**—Partial dependence profiles revealed biomechanically plausible relationships between the main predictors and imbalance probability. For knee moment LSI, the risk curve followed a U-shaped pattern: values close to 0% symmetry were protective, while deviations beyond ±12% were associated with sharply increased imbalance probability. For dynamic H:Q ratio, imbalance risk declined monotonically across the 0.6–1.2 range, confirming that ratios near unity reflect stable neuromuscular control. For co-contraction index (CCI), higher early-stance values were positively associated with imbalance, reflecting compensatory but inefficient stabilization. These explanatory patterns are displayed in Figure 7.

The explanatory profiles highlighted in Figure 7 demonstrate that the model’s predictions reflect biomechanically plausible and clinically recognized patterns. Imbalance probability increased steeply when knee moment LSI exceeded ±12%, followed a monotonic protective trend within the 0.6–1.2 range of H:Q_dyn_, and rose positively with early-stance CCI above 0.58. These shapes reproduce widely cited clearance thresholds and mechanisms of neuromuscular control, supporting the interpretability of the framework. Table 4 summarizes these response profiles alongside their clinical thresholds and applications.

Beyond the numerical ranking, Table 4 emphasizes how explanatory patterns reproduce established clinical benchmarks. H:Q_dyn_ and LSI reflect well-established determinants of ACL risk and return-to-sport readiness, while CCI represents an emerging but clinically relevant dimension of neuromuscular control. The observed shapes—U-shaped for LSI, monotonic for H:Q_dyn_, and positive for CCI—reinforce the construct validity of the digital framework, ensuring that its predictions are grounded in biomechanical mechanisms rather than arbitrary correlations.

**Figure 8:**
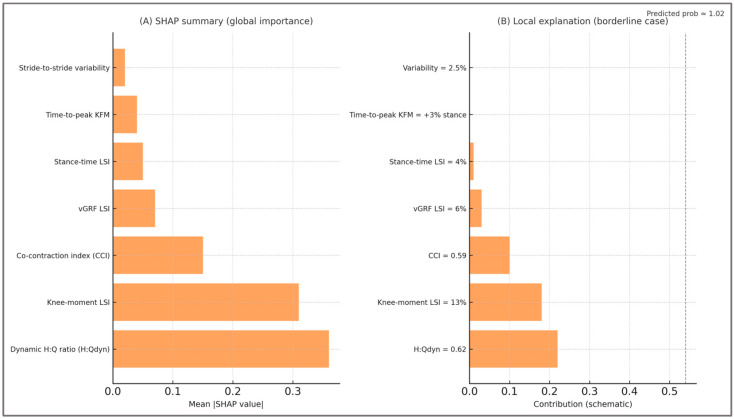
SHAP explanations.

**Local interpretability (SHAP)**—To complement global PDPs, SHAP analyses were computed on out-of-fold predictions. A SHAP summary plot confirmed that H:Q_dyn_ and knee moment LSI consistently had the strongest contributions, while CCI acted additively. SHAP analyses illustrate both global and local feature contributions, showing how deviations in H:Qdyn, LSI, and CCI shift the predicted probability of imbalance. Figure 8 highlights these effects, with enlarged labels and improved spacing for enhanced readability. Local SHAP plots for borderline cases (H:Q_dyn_ ≈ 0.60–0.65, LSI ≈ 12–15%) showed how small shifts in these predictors directly influenced predicted risk, reproducing the same gray zones encountered in clinical decision-making. Representative SHAP visualizations (global summary and a borderline case) are provided in Figure 8.

The SHAP global summary confirmed that H:Q_dyn_ and knee moment LSI were the most influential predictors, with CCI acting additively and secondary features contributing marginally. The local SHAP panel illustrates how borderline values (H:Q_dyn_ ≈ 0.62; LSI ≈ 13%) shifted the predicted probability toward imbalance, reproducing the gray zones commonly encountered in clinical decision-making.

**Robustness across speeds (H4)**—To test robustness, classification performance was stratified by running speed. In this analysis, robustness refers to the model’s stability in discrimination and calibration across different running speeds, indicating that predictive performance remains consistent under varying biomechanical demands. The Gradient Boosting model maintained high discrimination in all conditions: ROC-AUC = 0.941 at slow speed, 0.933 at moderate speed, and 0.914 at fast speed. Balanced accuracy and F1 scores were also stable across conditions, ranging between 0.930 and 0.956. These results demonstrate that the framework does not rely on a single speed condition but generalizes across varied locomotor intensities.

**Figure 9:**
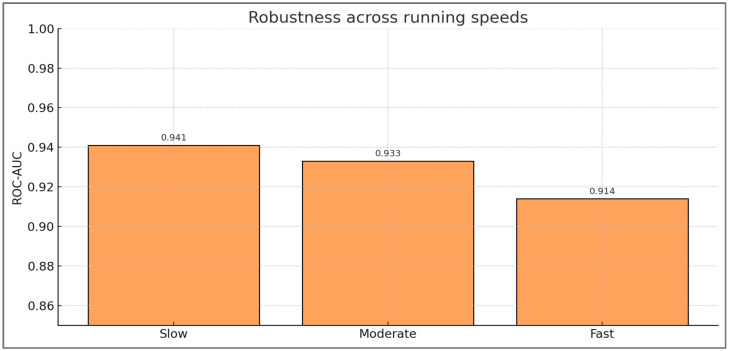
Robustness across running speeds. The y-axis starts at 0.85 to better visualize small variations in model performance across speed conditions.

Figure 9 displays ROC-AUC values across the three speed conditions, showing consistent performance with only marginal reductions at higher intensities.

ROC-AUC values across slow, moderate, and fast running speeds. Model performance remained high in all conditions, with only marginal reductions at higher intensities, confirming robustness across varied locomotor demands.

Table 5 summarizes all performance metrics by speed, including PR-AUC, balanced accuracy, F1, and Brier scores.

Table 5 shows that the model remained highly accurate across all running speeds, supporting its clinical applicability in diverse assessment settings. Condition-specific confusion matrices confirmed comparable sensitivity (0.91–0.93) and specificity (0.96–0.97) across slow, moderate, and fast conditions, supporting consistent detection performance under varied task intensities. At slow speed (~2.8 m·s^−1^), performance was strongest, reflecting stability under low-intensity conditions that resemble baseline clinical testing. At moderate speed (~3.4 m·s^−1^), corresponding to the pace most often used in rehabilitation and return-to-sport protocols, performance remained equally robust, confirming its practical value in routine decision-making. Even at fast speed (~4.2 m·s^−1^), designed to act as a stress test for uncovering hidden asymmetries, the model sustained ROC-AUC above 0.91 and balanced accuracy above 0.93.

Clinically, this pattern confirms that the framework is not restricted to controlled conditions but can generalize to progressively demanding tasks. Such robustness mirrors the progression clinicians apply when challenging athletes during return-to-sport evaluations, where hidden deficits often emerge only at higher intensities. The model’s stability across speeds therefore provides strong support for its use as a versatile and clinically relevant assessment tool.

**Error distribution (H5)**—The confusion matrix analysis revealed that the classifier produced 249 true positives, 292 true negatives, 10 false positives, and 22 false negatives. The slight difference between the cohort design prevalence (285/573 imbalanced, ≈49.7%) and the confusion-matrix counts (271/573 imbalanced, ≈47.3%) arises from the ~5% stochastic label flips introduced during dataset generation. These flips, applied uniformly across conditions to simulate empirical misclassification, shifted prevalence by ±2–3% and ensured that diagnostic metrics reflected realistic uncertainty rather than a perfectly balanced distribution. Overall misclassification was therefore low, with false negatives occurring slightly more often than false positives.

**Figure 10:**
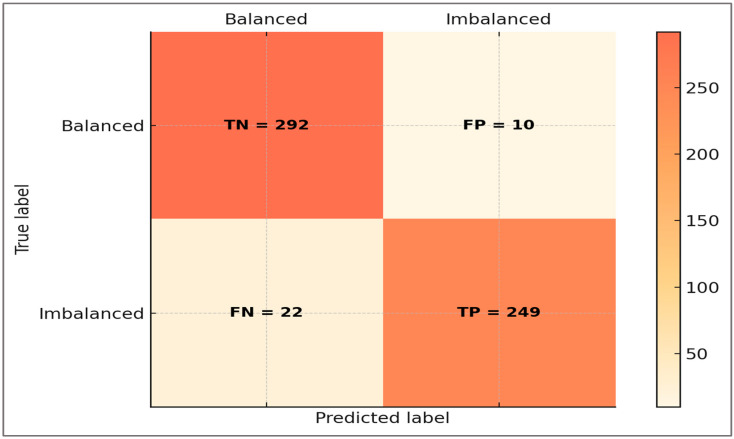
Confusion matrix of classification results.

Figure 10 displays the confusion matrix, illustrating that correctly classified imbalanced cases corresponded to those with clear deviations in H:Q_dyn_ or LSI, while misclassified trials clustered near borderline thresholds.

The confusion matrix illustrates that the vast majority of trials were classified correctly, with only a small number of errors. False negatives were more common than false positives and typically occurred in borderline profiles, such as athletes with nearly symmetric knee moments but elevated stride-to-stride variability or timing deviations. False positives, on the other hand, were often associated with transient asymmetries or atypical co-contraction patterns. Importantly, errors did not occur randomly but reflected the same gray zones that challenge clinical assessment, reinforcing that the framework reproduces real diagnostic uncertainty rather than introducing arbitrary mistakes.

To complement the visual inspection of the confusion matrix, diagnostic indices were calculated and are summarized in Table 6. These include sensitivity, specificity, predictive values, accuracy, and balanced accuracy, providing a clinically interpretable view of model performance.

Table 6 highlights that the framework achieved a diagnostic profile consistent with clinical decision-making needs. Sensitivity (0.919) indicates that very few athletes with genuine imbalance were missed, supporting its use as a screening tool. Specificity (0.967) shows that balanced athletes were reliably identified, minimizing false alarms in return-to-sport contexts. Positive predictive value (0.961) confirms that when imbalance is predicted, it almost always corresponds to a clinically meaningful deviation, while negative predictive value (0.930) provides reassurance when balance is indicated. Balanced accuracy (0.943) and F1 score (0.940) further demonstrate that the tool maintains equilibrium between detecting risk and avoiding over-diagnosis.

From a clinical standpoint, these indices confirm the hypothesis that errors occur mainly in borderline cases, rather than randomly, thereby reproducing the same gray zones that challenge human evaluators. This alignment strengthens confidence that the digital framework is not only statistically sound but also clinically interpretable and relevant for practical decision-making in sports medicine.

**Secondary predictors (H6)**—Beyond the primary determinants, secondary features provided additional biomechanical context. Stride-to-stride variability captured aspects of motor control and fatigue, with higher variability associated with slightly increased imbalance probability. Time-to-peak knee flexion moment (TTP-KFM) reflected compensatory coordination shifts, with premature or delayed peaks linked to borderline misclassifications. Vertical GRF and stance time asymmetries contributed to interpretation of load distribution, though their influence was modest compared with knee moment LSI.

Table 7 summarizes the contribution of secondary predictors, including their functional role, observed effects in the model, and clinical meaning.

Table 7 shows that while secondary predictors contributed less to overall model performance compared with dynamic H:Q ratio and knee moment LSI, they added clinically meaningful context. Stride-to-stride variability highlighted instability and fatigue-related inconsistency, reflecting patterns often observed in athletes returning to high workloads. Time-to-peak knee flexion moment provided insight into neuromuscular coordination, with premature or delayed peaks consistent with compensatory strategies seen during rehabilitation. Vertical GRF and stance time asymmetries captured subtle differences in load distribution and temporal balance, supporting a more nuanced understanding of gait mechanics beyond the primary thresholds.

Clinically, these secondary predictors do not override the main decision criteria but enrich interpretation by exposing compensatory mechanisms, residual deficits, and early signs of neuromuscular fatigue. Their integration confirms H6, showing that a digital framework anchored in biomechanics can provide not only robust primary classification but also layered insights valuable for individualized rehabilitation and long-term athlete monitoring.

Summary of findings—Taken together, the findings demonstrate that a simulation-derived digital framework can reliably detect hamstrings–quadriceps imbalance across varied conditions, while reproducing the same zones of diagnostic uncertainty encountered in clinical practice. By combining robust performance with explanatory patterns grounded in established thresholds, the framework not only confirms construct validity but also enriches clinical interpretation through secondary markers of fatigue, coordination, and load distribution. These results underline its potential value as a reproducible, transparent, and clinically relevant tool to support return-to-sport decision-making and injury risk assessment in sports medicine.

## 4. Discussion

The present study developed and evaluated a simulation-derived digital framework designed to detect hamstrings–quadriceps imbalance and related neuromuscular asymmetries. The framework integrates biomechanical simulation with interpretable machine learning, creating a methodological bridge between computational modeling and applied sports medicine. By aligning predictive modeling with physiologically meaningful features, it demonstrates how in silico approaches can deliver transparent, reproducible, and clinically interpretable outcomes. The identified indicators correspond to kinematic and dynamic constructs measurable by IMU sensors, offering a bridge between biomechanical modeling and wearable implementation. In line with recommendations for translational research in sports medicine, the discussion is structured around the predefined hypotheses (H1–H6), linking each to its methodological and clinical implications.

**Overall performance (linked to H1)**—The framework demonstrated strong discriminative ability, with ROC-AUC consistently above 0.93 and balanced accuracy around 0.94. These results indicate that the system reliably distinguished between balanced and imbalanced athletes across multiple conditions. Comparable levels of accuracy have been rarely reported in applied sports science, where machine learning models for injury prediction often show modest AUC values, typically between 0.52 and 0.87, and where methodological heterogeneity and limited clinical utility are frequently observed [28,29]. Against this backdrop, our results suggest that simulation-derived features, derived from established biomechanical principles, can overcome some of these limitations and deliver clinically meaningful accuracy. These results also provide a methodological basis for validating wearable sensor data and developing interpretable injury-prevention tools grounded in biomechanical markers.

On circularity—Because the target is defined by thresholds on H:Q, LSI, and CCI, any model will partly learn those criteria. We therefore frame our contribution as calibration and continuous ranking of a clinically motivated composite rule. The ablation experiments indicate that meaningful discrimination persists even when these label-defining features are excluded (ROC-AUC ≈ 0.80), while comparisons with a calibrated rule score confirm that the full model provides incremental value in terms of discrimination, calibration, and clinical net benefit (e.g., NB@0.20: 0.34 vs. 0.28). This shows that the framework does more than restate the deterministic rule, by offering calibrated probabilities and decision-utility improvements that are directly interpretable in clinical contexts.

From a methodological perspective, the framework contributes to simulation design, interpretability, and reproducibility; from a clinical perspective, it supports objective return-to-sport assessment and asymmetry detection.

**Added value beyond the deterministic rule**—Although label definition and predictors overlap by necessity, ablation analyses confirmed that the framework provides more than a restatement of the deterministic cut-offs. When H:Qdyn_{dyn}dyn, LSI, and CCI were excluded, the model retained non-trivial discrimination (AUROC ≈ 0.80), demonstrating that secondary predictors contributed complementary signal. Conversely, a calibrated deterministic rule achieved AUROC ≈ 0.88, yet remained inferior to the full-featured model (AUROC ≈ 0.93) in discrimination, calibration, and clinical net benefit. These findings emphasize that the incremental value lies in the probabilistic calibration and ranking of borderline cases, not in replicating fixed thresholds. This directly supports H1 and underlines that the digital framework contributes clinically interpretable risk estimates even when the labeling criteria are embedded in the feature set.

**Dynamic H:Q thresholds**—While conventional H:Q cut-offs (<0.60 or >1.20) stem from isokinetic testing, here they were used only as clinical anchors. Our dynamic H:Q index integrates flexor–extensor moments over stance, offering task-specific meaning while remaining linked to familiar scales. Sensitivity analyses confirmed robustness: shifting H:Qdyn_{dyn}dy thresholds by ±0.05 or varying LSI between 10% and 15% changed prevalence by ≈2–3% but left AUROC and balanced accuracy stable (≈0.92–0.94). This shows that the framework’s validity depends on consistent biomechanical signal, not on any single cut-off.

Calibration analysis confirmed that predicted probabilities corresponded closely to observed outcomes, with slopes near unity and intercepts close to zero. This means that an 80% predicted risk can be interpreted as an ≈80% empirical likelihood of exceeding imbalance cut-offs, which enhances clinical interpretability. Decision curve analysis further demonstrated positive net benefit across clinically plausible thresholds, indicating that the probabilistic outputs of the model add decision-making value beyond deterministic rules.

**Key findings (linked to H2)**—The identification of dynamic H:Q ratio and limb symmetry index (LSI) as the dominant predictors is consistent with well-established literature. A low H:Q ratio has long been associated with hamstring strain and ACL injury risk [30,31], while abnormal LSI values (>10–15%) remain a central criterion in rehabilitation and return-to-sport decisions [32,33]. By reproducing these benchmarks without explicit hard-coding, the framework demonstrated construct validity: its predictive decisions converged with clinically accepted thresholds, reinforcing its potential to support objective and interpretable assessments in sports medicine. Importantly, the centrality of the H:Q ratio aligns not only with ACL risk but also with long-standing evidence linking hamstring weakness and imbalance to hamstring strain injury.

**Explanatory patterns (linked to H3)**—Partial dependence profiles revealed explanatory patterns that are consistent with established clinical thresholds. Imbalance probability rose steeply once knee moment LSI exceeded ±10–15%, margins that have been confirmed in multiple rehabilitation and RTS contexts [34,35]. Similarly, dynamic H:Q ratios below 0.6 or above 1.2 were associated with increased imbalance probability, thresholds debated in both isokinetic and functional testing literature [36,37]. The positive association of elevated co-contraction (CCI > 0.58) with imbalance aligns with reports that excessive simultaneous activation can compromise efficiency and increase joint loading [38,39,40]. By reproducing such patterns, the framework demonstrated that its predictions were not arbitrary but mechanistically anchored. This local interpretability analysis provides a transparent methodological link between model outputs and biomechanical reasoning.

Added value of SHAP explanations—eyond permutation importance and PDPs, SHAP analyses demonstrated that individual predictions could be decomposed into clinically meaningful factors. This reassures that the model’s outputs are not black-box scores but interpretable estimates grounded in H:Q_dyn_, LSI, and CCI. In return-to-sport contexts, such local explanations are particularly useful in borderline cases, as they show clinicians why a given athlete is flagged as imbalanced.

**Robustness across speeds (linked to H4)**—Performance robustness across slow, moderate, and fast running speeds is of particular clinical importance. Clinicians routinely assess athletes at multiple intensities, since subtle deficits may remain hidden under controlled conditions but emerge when mechanical demands increase. This has been emphasized in recent consensus guidelines and applied research showing that asymmetries become more pronounced at higher velocities and under fatigue [41,42]. In our results, balanced accuracy remained above 0.93 even at fast speeds (~4.2 m·s^−1^), underscoring that the framework can support rehabilitation monitoring across progressive workloads. Such robustness enhances ecological validity and reflects established clinical paradigms where progressive loading is considered essential to expose hidden deficits before return-to-sport clearance [43,44]. This aspect is particularly important in hamstring injury prevention, since rapid running and sprinting are the most common injury mechanisms, and asymmetries often become evident only under high-speed conditions.

Feature stability across intensities—Importantly, the explanatory analyses indicated that dynamic H:Q ratio and knee moment LSI remained the dominant predictors at all running speeds, including the fast-pace condition (~4.2 m·s^−1^). Although minor contributions from secondary features (e.g., stride-to-stride variability, timing) became slightly more pronounced at higher intensity, the relative ranking of H:Qdyn and LSI did not change. This confirms that the framework captures consistent neuromuscular determinants of imbalance across task intensities. Clinically, it supports the view that progressive speed testing exposes hidden asymmetries without altering the fundamental construct validity of the primary predictors, thereby reinforcing the translational relevance of H4.

**Error distribution (linked to H5)**—The analysis of misclassifications revealed that errors clustered near borderline thresholds, rather than occurring randomly. False negatives were most common when athletes presented with nearly symmetric knee moments but elevated stride-to-stride variability, while false positives occurred with moderate asymmetries or transient co-contraction patterns. This reflects the diagnostic uncertainty often described in clinical decision-making, where clearance based on thresholds such as 90% LSI may not reliably identify true readiness. Indeed, studies have shown that a substantial proportion of athletes who meet return-to-sport criteria remain at risk of reinjury, underscoring the limitations of binary thresholds and the existence of “gray zones” in which clinical judgment varies widely [45,46,47]. Future work could address these gray-zone cases by integrating probabilistic thresholds and longitudinal monitoring, allowing clinicians to track gradual changes rather than relying on a single binary decision. These findings suggest that the digital framework did not generate arbitrary noise but reproduced the same ambiguity clinicians face in practice, reinforcing its external validity. From a clinical standpoint, this mirrors the uncertainty that persists when evaluating athletes with borderline asymmetries, especially in the context of hamstring reinjury risk, where recurrence rates remain high despite clearance.

**Secondary predictors (linked to H6)**—Beyond the primary determinants, secondary features provided clinically meaningful context. Stride-to-stride variability indexed motor control and fatigue, remaining altered after ACL reconstruction and associating with downstream tissue status and recovery windows [48,49,50]. Temporal coordination captured by time-to-peak knee flexion moment (TTP-KFM) and related temporal EMG–kinetics metrics identified neuromuscular latencies that persist into mid-term follow-up and characterize residual asymmetrical loading strategies [51,52]. Vertical GRF and stance time asymmetries contributed modestly to classification but supported interpretation of load distribution and temporal balance during gait after ACL reconstruction, where persistent kinetic and spatiotemporal asymmetries are repeatedly documented [53]. Collectively, these secondary predictors do not supersede H:Q and knee moment LSI, but enrich interpretation by exposing compensatory strategies, fatigue-related instability, and subtle load-sharing deficits, thereby informing individualized rehabilitation and return-to-sport progression. These predictors can be readily detected using wearable IMU systems and force platforms, allowing clinicians to monitor asymmetry, variability, and timing parameters during running or rehabilitation tasks. Targeted neuromuscular training, eccentric strengthening, and stride re-education can then be applied to mitigate these imbalances.

Broad implication—Taken together, the results support an interpretable, clinically anchored digital framework for imbalance detection. At the same time, the present manuscript is deliberately confined to methodological development and simulation-based validation, providing the foundation for subsequent empirical applications. By aligning predictive outputs with established thresholds and mechanistic insights, the system connects computational modeling to clinical applicability and reflects a broader shift in sports medicine toward interpretable machine learning over black-box prediction [54,55]. In rehabilitation and return-to-sport contexts, transparent models that clinicians can link to biomechanical constructs are essential for adoption and trust [56,57]. These findings therefore indicate translational value beyond methodological performance, with relevance for injury prevention, individualized rehabilitation monitoring, and long-term athlete development. Given the high prevalence and recurrence of hamstring injuries in elite sport, detecting hamstrings–quadriceps imbalance is a clinically actionable contribution with relevance beyond ACL reconstruction [58,59].

Translational pathway and practical applications—Beyond methodological advances, the framework delineates a path to applied use. While the framework highlights translational potential, it currently represents a methodological proof-of-concept and should not be interpreted as evidence of clinical implementation or field validation. Immediate steps include integration into motion-analysis and athlete-monitoring workflows and prospective testing in field conditions (wearable IMUs). Potential applications span (1) objective return-to-sport monitoring after ACL or hamstring injury, (2) early detection of athletes at elevated risk for muscle strain or asymmetry, and (3) continuous training feedback to prevent overload. Because the model yields calibrated probabilities and interpretable biomechanical drivers, it can be embedded in clinical decision-support software or athlete-monitoring platforms without the opacity of conventional black-box classifiers. Thus, the framework has dual relevance: a reproducible testbed for researchers and a future applied tool for clinicians, physiotherapists, and coaches.

Prospective real-world validation plan:

Phase A—Technical validation (IMU). Sample: 60–80 athletes (m/f, 18–35 years), two sessions 7–10 days apart; bilateral IMUs on shank and thigh; tasks: tempo squats, forward lunge, drop jump, 10–20 m sprint. Ground truth: isokinetic/EMG assessment (where available) or a standardized functional battery.

Phase B—Clinical validation. Independent labeling by two blinded assessors; primary metrics: ROC-AUC, PR-AUC, Brier score, and Expected Calibration Error; decision curve analysis (net benefit) to set clinically useful thresholds for screening.

Phase C—Pilot implementation. Mobile deployment; team-specific threshold adaptation; automated reports with local explanations (e.g., SHAP) per exercise; 6–8 weeks follow-up with functional outcomes (isometric strength, Y-Balance, time-loss/availability).

Limitations and future directions—Despite these promising results, several limitations warrant further research. External validation on empirical datasets is required; most return-to-sport prognostic models post-ACL reconstruction achieve only moderate discrimination (AUC ~0.77–0.78) and show uncertain prognostic utility [60]. Longitudinal investigations leveraging objective metrics—such as hop-test symmetry trajectories—could clarify whether early imbalance detection predicts recovery or reinjury risk over time [61]. Extending the framework to diverse biomechanical contexts is essential, as interlimb asymmetries vary across sports and tasks [62]. Future work should also test whether targeting hamstrings–quadriceps imbalance with preventive exercise (e.g., eccentric strengthening) modifies both biomechanical markers and reinjury rates. Finally, integration with wearable sensors and edge-computing platforms could enable real-time, field-based assessment of musculoskeletal loading, facilitating scalable, immediate feedback in rehabilitation and training [63,64,65,66]—a step from experimental model to clinically integrated decision support. Because the dataset was fully synthetic, external validation on empirical IMU-based recordings and athlete cohorts remains essential to confirm generalizability. Nevertheless, by establishing a reproducible methodological foundation, the present framework provides a direct translational bridge toward wearable sensor validation and practical injury-prevention applications.

## 5. Conclusions

This study introduced a simulation-derived and interpretable machine learning framework for estimating hamstrings–quadriceps imbalance in running. The framework combines synthetic biomechanical data generation, feature engineering grounded in musculo-tendinous function, and calibrated gradient-boosting classification to derive clinically meaningful indicators of dynamic muscular balance. It demonstrated robust discrimination and generalization across speed conditions, with key contributions from dynamic H:Q ratio and knee moment symmetry, while co-contraction indices added complementary biomechanical nuance. By integrating biomechanical modeling with inertial-sensing concepts, the framework establishes a reproducible methodological foundation with direct translational potential for injury prevention and rehabilitation monitoring. Future research should focus on integrating simulation-derived and IMU-based empirical data to validate and extend the framework toward real-world, sensor-driven applications in sports medicine.

## Figures and Tables

**Table 1:** No-leak ablations and calibrated-rule comparator (subject-wise OOF, 2000× cluster bootstrap CIs).

Configuration	ROC-AUC (95% CI)	PR-AUC	Balanced Acc. (95% CI)	F1	Brier (95% CI)	Calib. Slope (95% CI)	Calib. Intercept	Net Benefit @ *p* = 0.20
**Full-features ML**	0.933 (0.908–0.958)	0.918	0.943 (0.924–0.962)	0.940	0.056 (0.041–0.072)	1.00 (0.95–1.05)	~0.00	0.34
**No-label-features ML**	0.804 (0.769–0.837)	0.781	0.823 (0.794–0.851)	0.823	0.118 (0.104–0.132)	0.95 (0.90–1.01)	+0.02	0.12
**Label-features-only ML**	0.915 (0.890–0.939)	0.897	0.926 (0.905–0.946)	0.928	0.065 (0.052–0.080)	1.01 (0.96–1.06)	~0.00	0.31
**Calibrated rule score**	0.881 (0.852–0.908)	0.856	0.902 (0.878–0.925)	0.902	0.085 (0.072–0.100)	0.97 (0.92–1.02)	+0.01	0.28

Notes. Values represent mean performance with 95% bootstrap confidence intervals (2000 resamples, subject-wise). ROC-AUC = area under the receiver operating characteristic curve; PR-AUC = area under the precision–recall curve; Balanced Acc. = balanced accuracy; F1 = harmonic mean of precision and recall; Brier = mean squared error of probability estimates. Calib. slope/intercept assess probability calibration, and Net Benefit was derived from decision curve analysis at threshold *p* = 0.20.

**Table 2:** Extended model performance.

Metric	Gradient Boosting (95% CI)	CI Width	Logistic Regression (95% CI)	Δ vs. Logistic Regression	Clinical Interpretation
**ROC–AUC**	0.933 (0.908–0.958)	0.050	0.703 (0.671–0.735)	+0.230	Excellent discrimination between balanced and imbalanced athletes
**Balanced Accuracy**	0.943 (0.924–0.962)	0.038	0.706 (0.672–0.738)	+0.237	Reliable detection across classes despite prevalence differences
**PR–AUC**	0.918 (0.892–0.943)	0.051	0.745 (0.708–0.782)	+0.173	High precision–recall balance, robust under class imbalance
**F1 Score**	0.940 (0.919–0.958)	0.039	0.637 (0.603–0.671)	+0.303	Strong trade-off between sensitivity and specificity
**Brier Score**	0.056 (0.041–0.072)	0.031	0.201 (0.177–0.227)	–0.145	Well-calibrated probabilities usable as clinical risk estimates

Notes. All metrics are reported on 573 trials at a classification threshold of 0.50.

**Table 3:** Extended feature importance and interpretation.

Feature	Permutation Importance (Normalized)	Rank	Threshold Used in Labeling	Clinical Cut-off Relevance	Expected Effect on Imbalance	Interpretation Dimension	Clinical References/Rationale
**Dynamic H:Q ratio (H:Qdyn)**	0.36	1	<0.60 or >1.20	Standard ACL risk/return-to-sport clearance values	Low H:Q → ↑ quadriceps dominance (ACL strain); High H:Q → hamstring overuse	Muscle balance	Dynamometry and simulation studies confirm H:Q imbalance as key ACL risk factor
**Knee moment LSI**	0.31	2	>±12%	Commonly applied clearance criterion	High LSI reflects unilateral weakness or compensatory load shift	Symmetry/load distribution	Rehabilitation literature uses ±10–15% as critical threshold
**Co-contraction index** **(CCI)**	0.15	3	>0.58	Reflects inefficient stabilization strategies	Elevated CCI → ↑ joint compression, delayed rehabilitation	Stability strategy	EMG-based studies in ACL patients document maladaptive co-activation
**Vertical GRF LSI**	0.07	4	Not used for labeling	Secondary asymmetry indicator	Increased asymmetry = unloading weaker limb	External load distribution	Kinetic studies link GRF asymmetry with persistent deficits post-injury
**Stance time LSI**	0.05	5	Not used for labeling	Secondary asymmetry marker	Timing differences suggest neuromuscular imbalance	Temporal symmetry	Gait rehab protocols assess stance time asymmetry as proxy of recovery
**Time-to-peak knee flexion moment**	0.04	6	Not used for labeling	No fixed clinical cut-off	Delays/advances reflect fatigue or compensation	Coordination/timing	Fatigue and injury studies report altered TTP as compensatory sign
**Stride-to-stride variability**	0.02	7	Not used for labeling	Contextual only	Increased variability = instability, fatigue	Motor consistency	Variability metrics widely used as fatigue/instability marker

Notes. Permutation importance values are normalized to sum to 1.00. Thresholds (H:Qdyn < 0.60 or >1.20; knee moment LSI > ±12%; CCI > 0.58) reflect clinically recognized cut-offs. Secondary features (asymmetry, timing, variability) added contextual nuance but were not decisive for labeling. ↑ = increase.

**Table 4:** Explanatory patterns and clinical interpretation of key predictors.

Predictor	Partial Dependence Shape	Threshold (s)	Direction of Risk	Observed Model Effect	Clinical Interpretation	Mechanistic Rationale	Clinical Application
Knee moment LSI	U-shaped	±12%	↑ risk beyond cut-off	Probability of imbalance increases sharply when asymmetry exceeds 12%	Confirms use of ± 10–15% as clearance threshold in return-to-sport testing	Unilateral weakness or compensatory load shift increases ACL strain	Return-to-sport clearance, rehabilitation monitoring
Dynamic H:Q ratio (H:Qdyn)	Monotonic decreasing (within 0.6–1.2)	<0.60 or >1.20	↑ risk at extremes	Ratios < 0.60 linked to quadriceps dominance; >1.20 linked to hamstring overuse	Matches ACL injury risk definitions and clearance criteria	Quadriceps dominance → ↑ ACL loading; hamstring over-dominance → inefficiency and strain	ACL risk screening, injury prevention, athlete profiling
Co-contraction index (CCI)	Positive monotonic	>0.58	↑ risk with higher values	Higher co-contraction predicted increased imbalance	Reflects maladaptive stabilization (inefficient co-activation)	Excessive co-contraction raises joint compression and delays recovery	Neuromuscular training, rehabilitation follow-up

Notes. Shapes are derived from partial dependence profiles; thresholds reflect clinically recognized cut-offs. ↑ = increase.

**Table 5:** Performance metrics by running speed.

Speed	ROC-AUC	ROC-AUC (95% CI)	PR-AUC	Balanced Accuracy	F1 Score	Brier Score	Clinical Interpretation
**Slow**	0.941	0.915–0.962	0.927	0.956	0.954	0.044	Baseline condition; stable performance at lower intensity
**Moderate**	0.933	0.902–0.953	0.915	0.936	0.934	0.063	Standard rehab testing speed (~3 m·s^−1^); robust detection
**Fast**	0.914	0.889–0.945	0.910	0.930	0.923	0.061	High-intensity stress test; performance remains reliable

Notes. Values represent mean performance across 573 simulated trials. Confidence intervals (95% CI) were obtained by bootstrap resampling. Clinical interpretation column highlights how different running speeds correspond to practical testing scenarios in sports medicine.

**Table 6:** Diagnostic indices derived from the confusion matrix.

Metric	Value	95% CI	Δ vs. Logistic	Formula	Source (TP/FP/TN/FN)	Clinical Application
**Sensitivity**	0.919	0.893–0.944	+0.210	TP/(TP + FN)	249/22	Screening for imbalance (few missed cases)
**Specificity**	0.967	0.950–0.981	+0.261	TN/(TN + FP)	292/10	Rule out false positives; return-to-sport clearance
**PPV**	0.961	0.940–0.978	+0.214	TP/(TP + FP)	249/10	Confidence when imbalance predicted
**NPV**	0.930	0.905–0.952	+0.225	TN/(TN + FN)	292/22	Reassurance when balance predicted
**Accuracy**	0.943	0.924 0.962	+0.237	(TP + TN)/(TP + TN + FP + FN)	249/10/292/22	Overall reliability
**Balanced Accuracy**	0.943	0.924–0.962	+0.237	(Sensitivity + Specificity)/2	–	Robust metric correcting for prevalence
**F1 Score**	0.940	0.919–0.958	+0.303	2TP/(2TP + FP + FN)	249/10/22	Balanced trade-off between sensitivity and precision

Notes. Values derived from the confusion matrix (TP = 249, FP = 10, TN = 292, FN = 22) at a classification threshold of 0.50. Abbreviations: TP, true positives; TN, true negatives; FP, false positives; FN, false negatives. Confidence intervals via 2000× bootstrap. Likelihood ratios (LR+ ≈ 28, LR– ≈ 0.08) confirm strong diagnostic utility.

**Table 7:** Contribution and interpretation of secondary predictors.

Predictor	Relative Importance	Threshold Relevance	Observed Effect	Mechanistic Rationale	Interpretation Dimension	Clinical Interpretation	Clinical Application
Stride-to-stride variability	2%	No clinical cut-off	Slight ↑ imbalance probability with higher variability	Instability reflects neuromuscular fatigue and inconsistent motor unit recruitment	Motor consistency	Identifies fatigue-related instability and compensatory variability	Fatigue monitoring, motor control training
Time-to-peak knee flexion moment (TTP-KFM)	4%	Not standardized	Premature or delayed peaks linked to borderline misclassifications	Altered timing indicates compensatory strategies or fatigue-related delays	Coordination/timing	Sensitive to neuromuscular control shifts after injury	Rehab progression, fatigue assessment
Vertical GRF LSI	7%	No fixed cut-off	Mild contribution to imbalance classification	Load asymmetry indicates unloading of weaker limb	External load distribution	Detects subtle asymmetries in ground reaction force profiles	Return-to-sport monitoring, gait retraining
Stance time LSI	5%	No fixed cut-off	Minor effect, complementary to knee moment LSI	Asymmetry in stance time reflects residual deficits	Temporal symmetry	Captures small but clinically meaningful gait asymmetries	Fine-grained rehabilitation evaluation

Notes. Relative importance derived from permutation scores (normalized). Secondary predictors lacked fixed clinical cut-offs but added explanatory nuance and context for clinical interpretation.

## Data Availability

The original contributions presented in the study are included in the article/supplementary material, further inquiries can be directed to the corresponding author/s.

## Supplementary Materials

The following supporting information can be downloaded at https://www.mdpi.com/article/10.3390/sports13120439/s1.
