# Supplementary material for: Interpretable Machine Learning on Simulation-Derived Biomechanical Features for Hamstrings–Quadriceps Imbalance Detection in Running
Source: Sports (Basel). 2025 Dec 5;13(12):439. doi: 10.3390/sports13120439 (PMC12736594; doi:10.3390/sports13120439)
Supplement: Supplementary file 1 [file sports-13-00439-s001.zip › sports-3942919-supplementary.pdf]

sports

MDPI

Interpretable Machine Learning on Simulation-Derived Biomechanical Features for Hamstrings–Quadriceps Imbalance Detection in Running

Andreea Maria Mănescu[2,*], Andrei Claudiu Tudor[1], Corina Claudia Dinciu[1], Simona Ștefania Hangu[1], Iulius Radulian Mărgărit[1], Virgil Tudor[3], Cătălin Octavian Mănescu[1], Rela Valentina Ciomag[1], Mihaela Loredana Rădulescu[1], Cristian Hangu[1], Neluța Smîdu[1], Victor Dulceață[1], Ioana Cosmina Barac[1], Sorin Cristian Niță[4], Carmen Grigoroiu[5], Dan Cristian Mănescu[1,*]

[1] Department of Physical Education and Sport, Bucharest University of Economic Studies, 010374 Bucharest, Romania
[2] Doctoral School, Faculty of AgriFood and Environmental Economics, Bucharest University of Economic Studies, 010374 Bucharest, Romania
[3] National University of Physical Education and Sport, 060057 Bucharest, Romania
[4] UNESCO Chair for Business Administration in Foreign Languages, Bucharest University of Economic Studies, 010374 Bucharest, Romania
[5] Department of Physical Education and Sport, National University of Science and Technology POLITEHNICA Bucharest, 060042 Bucharest, Romania
* Correspondence: dan.manescu@defs.ase.ro

Supplementary Materials

S1. Extended Methods

S1.1 Synthetic Data Generation

The first stage of the framework consisted of synthetic data generation, designed to emulate the structure and variability of athletic cohorts in a controlled digital environment. No experimental or human data were collected or analyzed; all datasets were generated synthetically within the simulation framework. Although no physical inertial sensors were used, the modeled segmental dynamics were structured to emulate IMU-level signal characteristics, ensuring translational compatibility with wearable-sensor data. This step combined virtual subjects with simulated running trials, introduced variability across three speed bands, and applied clinically anchored labeling rules based on H:Qdyn, LSI, and CCI. Together, these components define a digital cohort representative of athletic variability and imbalance patterns.

Virtual Subjects—A virtual cohort of 160 synthetic subjects was constructed to emulate the neuromuscular diversity of an athletic population. Each subject was assigned a latent imbalance propensity, sampled from a bimodal Gaussian distribution, creating two subpopulations representing balanced and imbalanced neuromechanical profiles. The decision to model imbalance at the *subject level*—rather than at the individual trial level—ensured that the virtual cohort reflected how real athletes are studied in biomechanics and

 https://doi.org/10.3390/xxxxx

sports medicine, where the participant remains the primary unit of analysis. This choice aligns the framework with clinical research practice, where repeated measurements are used to characterize the neuromuscular profile of each athlete rather than being treated as independent observations.

Methodological and Clinical Relevance—Subject-level design was further justified by the clinical importance of hamstrings–quadriceps (H:Q) imbalance in sports medicine. Prior studies have shown that H:Q ratios below ~0.6 or above ~1.2, together with limb asymmetries exceeding 10–15%, are predictive of heightened anterior cruciate ligament (ACL) injury risk and delayed return-to-sport clearance. By embedding these thresholds as latent parameters within each synthetic subject, the virtual cohort mirrored the decision criteria applied in rehabilitation and performance testing. This construction allowed the framework not only to simulate data but also to reflect the conceptual structure of clinical assessments, where imbalance is diagnosed per athlete, not per isolated trial.

Trials and Task Conditions—For each subject, between two and five running trials were simulated across three task intensities: slow (≈2.8 $m \cdot s^{-1}$), moderate (≈3.4 $m \cdot s^{-1}$), and fast (≈4.2 $m \cdot s^{-1}$). These speeds were chosen to correspond to experimental protocols in running biomechanics, where moderate velocities (~3 $m \cdot s^{-1}$) are typically used for baseline testing, and higher velocities (>4 $m \cdot s^{-1}$) reveal compensatory mechanisms that may not be apparent at lower intensities. A total of 573 trials were generated, producing a dataset large enough to evaluate model generalizability while maintaining subject-level coherence. This design ensured realistic intra-individual variability consistent with repeated-measures protocols in sports biomechanics.

Variability and Realism—Inter-individual heterogeneity was introduced by varying the latent imbalance parameters across subjects, thereby representing population-level diversity. Within each trial, stride-to-stride variability was modeled by perturbing biomechanical outputs, reflecting natural fluctuations in ground reaction forces, knee joint moments, and muscle activations. In addition, ~5% of labels were randomly flipped to simulate measurement errors and misclassification, which are common in empirical biomechanics due to sensor noise, marker placement variability, and EMG cross-talk. These design elements ensured that the synthetic dataset preserved not only structure but also the imperfections of real-world cohorts, avoiding the misleading stability of purely deterministic models.

Ethical and Methodological Advantages—The use of virtual subjects offered methodological advantages that extend beyond convenience. It allowed systematic manipulation of imbalance prevalence and severity without ethical risks associated with overloading real athletes. It also enabled precise control over confounding variables, such as trial intensity and noise level, which are difficult to isolate in empirical settings. Moreover, the digital cohort facilitated full reproducibility, since every subject could be regenerated under the same probabilistic rules, an advantage rarely achievable in clinical research where recruitment and data collection are subject to variability and attrition.

Taken together, the construction of the virtual cohort provided a physiologically plausible and methodologically transparent foundation for the in silico study. By grounding imbalance at the subject level, embedding variability at multiple scales, and aligning parameters with thresholds widely discussed in sports medicine, the framework ensured that

subsequent analyses retained direct muscular meaning. This approach positioned the synthetic cohort not as a statistical abstraction but as a controlled analogue of real athletes, in which hamstrings–quadriceps balance could be studied with rigor, reproducibility, and translational relevance.

Key design parameters of the virtual cohort, together with their methodological rationale and quantitative implementation, are summarized in Table S1.

Table S1. Overview of the virtual cohort design with methodological rationale, realism strategies, and quantitative parameters

Parameter	Implementation	Purpose / Rationale	Clinical / Biomechanical relevance	Data realism strategy	Numerical details (per subject / trial)	Aggregate stats (cohort-level)
**Subjects**	160 synthetic individuals, bimodal latent imbalance distribution	Balanced vs. imbalanced subpopulations; unit of analysis = subject	Mirrors athletes with vs. without imbalance	Gaussian bimodal sampling anchored to H:Q-informed imbalance	80 balanced, 80 imbalanced	160 subjects total
**Trials per subject**	2–5 trials, 3 running speeds	Captures intra-individual variability	Reflects repeated-measures protocols in biomechanics/rehab	Randomized trial count per subject	Median = 3; Mean ≈ 3.58; Range = 2–5	573 trials total
**Speeds**	Slow, Moderate, Fast	Tests imbalance across task intensities	Aligns with lab protocols (~3 and ~4 m·s^{-1})	Added stride-to-stride noise on target velocity	2.8 ± 0.1; 3.4 ± 0.1; 4.2 ± 0.1 m·s^{-1}	191 trials/condition
**Variability**	Inter-individual + stride-to-stride	Represents population and gait heterogeneity	Matches variability in kinetics/EMG	Multiplicative trial-wise perturbations	CV inter-individual ≈ 12%; intra-trial ≈ 5%	—
**Noise**	~5% random label flips	Simulates misclassification & measurement error	Reflects empirical uncertainty	Random label reassignment on OOF labels	≈ 29 flips total; by speed: 10 slow, 9 moderate, 10 fast	≈5% of 573 trials affected
**Class distribution by speed**	Balanced vs. imbalanced per condition	Ensures comparable prevalence across speeds	Avoids confounding by task intensity	Stratified generation per condition	Slow = 96/95; Moderate = 96/95; Fast = 96/95 (balanced/imbalanced)	Balanced = 288; Imbalanced = 285
**Label thresholds**	H:Q <0.6, LSI >10–15%, CCI ↑ early stance	Defines imbalance prevalence and severity	Corresponds to ACL risk and return-to-sport criteria	Threshold-based labeling with controlled prevalence shift	LSI cut-off 10%→15% = −1.8% prevalence; H:Q shift −0.05 = +2.1%	Net effect ≈ ±2–3% imbalance prevalence
**Aggregate output**	Generated synthetic dataset	Provides reproducible in silico cohort	Transparent methodological testbed	Seed-controlled generator	—	573 labeled trials total

Notes Prevalence shifts indicate the sensitivity of imbalance classification to threshold definitions (H:Q ratio, LSI, CCI). Small variations in cut-offs alter class prevalence by ~2–3%, underscoring the methodological link between digital labeling rules and clinical decision criteria.

Taken together, the construction of the virtual subjects provided a physiologically plausible and methodologically rigorous foundation, ensuring that subsequent running trials and labeling procedures remained anchored in hamstring–quadriceps physiology.

Running Trials—To complement subject-level design, each virtual individual was modeled through repeated running trials across multiple task intensities, ensuring that imbalance was expressed under dynamic and variable biomechanical conditions.

Trial design across three speed—Each virtual subject was simulated to perform between two and five running trials, spanning three standardized speed bands: slow (~2.8 $m \cdot s^{-1}$), moderate (~3.4 $m \cdot s^{-1}$), and fast (~4.2 $m \cdot s^{-1}$). These velocities were selected to reflect commonly adopted benchmarks in gait and sports biomechanics, where moderate speeds provide baseline neuromuscular assessment, and higher speeds elicit compensatory strategies that are often associated with elevated injury risk. The design yielded a total of 573 trials, distributed evenly across the three conditions, thereby ensuring statistical balance while maintaining subject-level variability.

Biomechanical relevance of running speed—Speed modulation is known to influence joint loading, muscle activation dynamics, and neuromechanical control strategies. At lower running speeds, joint kinetics are more symmetrical and less demanding, whereas faster speeds amplify asymmetries and challenge the hamstrings–quadriceps balance by increasing extensor torque requirements and co-contraction demands. Embedding this range of velocities into the virtual cohort ensured that imbalance was evaluated not under static or idealized conditions, but across ecologically valid task intensities that replicate the challenges of sports performance.

Intra-trial stride-to-stride variability—To enhance realism, stride-to-stride variability was introduced within each simulated trial. Instead of producing identical repetitions, small perturbations were added to ground reaction forces, joint moments, and activation timing, reproducing the natural cycle-to-cycle variability observed in running biomechanics. This design captures neuromuscular noise and motor-unit recruitment variability typical of human movement, preventing the unrealistic stability of purely deterministic simulations.

Methodological and clinical significance—The inclusion of both speed variation and stride variability served not only to increase dataset diversity but also to reinforce clinical and methodological relevance. In experimental biomechanics, repeated trials across different speeds are a cornerstone of return-to-sport assessment, as they expose hidden asymmetries and neuromuscular deficits that may not be evident under controlled, low-intensity tasks. By integrating these principles into the in silico framework, the virtual running trials provided a physiologically grounded substrate from which biomechanical features could be extracted, ensuring that subsequent analyses retained direct muscular and clinical meaning.

Taken together, the structure of repeated running trials across different speeds, enriched with stride-to-stride variability, provided a realistic experimental substrate for generating biomechanical features and defining imbalance in a clinically meaningful manner.

Labeling Strategy—To transform raw simulations into clinically interpretable outcomes, each trial was assigned a class label based on established biomechanical thresholds, thereby defining balanced versus imbalanced conditions.

Thresholds and biomechanical rationale—Labels were assigned according to three core constructs: the dynamic hamstrings–quadriceps ratio ($H{:}Q_{dyn}$), the knee moment limb symmetry index (LSI), and the co-contraction index (CCI) during early stance. Thresholds were

set at $H:Q_{dyn}$ <0.6 or >1.2, LSI >10–15%, and elevated CCI beyond the expected physiological range, reflecting benchmarks commonly cited in sports medicine. These cut-offs were selected because they correspond to return-to-sport clearance criteria and predictors of anterior cruciate ligament (ACL) injury risk, thereby anchoring the labeling system in clinically recognized definitions of imbalance.

Integration into the synthetic framework—The thresholding rules were embedded directly into the synthetic cohort at the subject level, ensuring that each virtual athlete was consistently classified across trials. For instance, a subject with systematically low H:Qdyn values would be designated imbalanced across all trials, while stride-level fluctuations around the threshold could still produce trial-to-trial variability. This approach reflected how athletes are clinically categorized: as balanced or imbalanced individuals, even though variability is inherent in repeated performance assessments.

Sensitivity to cut-offs and prevalence shifts—Because imbalance prevalence depends on threshold selection, systematic sensitivity analyses were conducted. Shifting the LSI cut-off from 10% to 15% reduced imbalance prevalence by ~1.8%, while a −0.05 adjustment to the $H:Q_{dyn}$ threshold increased prevalence by ~2.1%. Overall, prevalence varied within ±2–3% across reasonable threshold ranges. These controlled shifts ensured that labeling was both realistic and transparent, demonstrating that clinical decision rules inherently carry uncertainty that propagates into classification outcomes.

Noise and realism — To simulate realistic measurement uncertainty, approximately 5 % of labels were randomly flipped to represent typical misclassification and evaluator error in biomechanics. This procedure introduced minor prevalence shifts of about ±2–3 %, reflecting how threshold variability and noise affect empirical datasets while preventing artificially perfect conditions.

Taken together, the labeling strategy anchored imbalance detection in clinically validated thresholds while acknowledging the variability and uncertainty inherent to empirical practice, thereby reinforcing the physiological credibility of the synthetic cohort.

In this way, the synthetic cohort established the conditions from which biomechanical features could be systematically extracted and analyzed.

Linking Dynamic H:Q to Clinical Cut-offs — Conventional hamstrings–to–quadriceps (H:Q) thresholds (e.g., <0.60 or >1.20) originate primarily from isokinetic dynamometry, which reflects isolated strength under static or joint-controlled conditions. In this proof-of-concept, these values were therefore used as clinical anchors rather than as literal transfer values. The imbalance definition operationalized here is based on a dynamic H:Q index (H:Qdyn_{dyn}dyn) that integrates flexor–extensor moments over the stance phase, thereby providing a task-specific reflection of knee loading during running. This design preserves the link with familiar clinical scales while recognizing that dynamic ratios may differ quantitatively from static measures.

To ensure that conclusions do not hinge on any single numerical choice, we performed pre-specified sensitivity analyses by shifting H:Qdyn_{dyn}dyn cut-offs by ±0.05 and varying the knee moment LSI threshold between 10% and 15%. These perturbations altered class prevalence by only ≈2–3% and confirmed that discrimination and calibration remained stable across plausible definitions. Thus, the framework should be interpreted as

anchored to clinically recognized thresholds, yet robust to their exact placement, reinforcing its validity as a methodological baseline for imbalance detection.

Transparency of the generator—To ensure reproducibility, the virtual cohort and trial structure were generated using explicit formulas and parameter values reported in Section 4.1. All stochastic elements, including Gaussian perturbations and ~5 % label flips, were controlled by a fixed random seed (seed = 2025), allowing the dataset to be regenerated identically from the provided specifications. This emphasizes methodological transparency: imbalance labels are clinically anchored ($H:Q_{dyn} < 0.60$ or > 1.20; $|LSI| > 12$ %; CCI > 0.58) with controlled variability and misclassification noise to mimic empirical conditions.

S1.2. Simulation-Derived Feature Set

Dynamic Hamstrings-to-Quadriceps Ratio ($H:Q_{dyn}$)—The H:Q ratio is a cornerstone metric for evaluating the balance between knee flexors (hamstrings) and extensors (quadriceps). Unlike static or isokinetic measures, the dynamic version integrates moments over the stance phase, providing a task-specific index of relative contribution:

$$H:Q_{dyn} = \frac{\int M_{flex}(t)\,dt}{\int M_{ext}(t)\,dt}$$

where $M_{flex}(t)$ and $M_{ext}(t)$ are the knee flexor and extensor moments, while dt indicates summation over infinitesimal time incrementsand. The interval t correspond to the stance phase (from foot-strike to toe-off). This measure reflects the total effort of hamstrings relative to quadriceps. To avoid numerical instability when extensor moments approached zero, the denominator was stabilized with $\varepsilon = 0.01$.

Conceptually, $H:Q_{dyn}$ values below ~0.6 indicate disproportionate quadriceps dominance, whereas values above ~1.2 reflect hamstring over-dominance. Both extremes have been repeatedly associated with anterior cruciate ligament (ACL) injury risk and with delayed return-to-sport clearance, making $H:Q_{dyn}$ a clinically meaningful indicator of neuromuscular imbalance.

Limb Symmetry Index (LSI)—LSI is a widely used metric in biomechanics and sports medicine to quantify inter-limb asymmetry. It expresses the relative difference between the dominant and non-dominant limb, normalized as a percentage:

$$LSI = 100 \cdot \frac{X_{dom} - X_{non-dom}}{0.5 \cdot (X_{dom} + X_{non-dom})} \backslash\%$$

where X_{dom} and $X_{non-dom}$ represent peak values of the same feature (e.g., knee moment, stance time, or vertical GRF) from the dominant and non-dominant limb. An LSI of 0% indicates perfect symmetry; positive values indicate dominance of the stronger limb, while negative values favor the weaker limb. Dominant vs. non-dominant limb assignment was fixed at the subject level across all trials to ensure consistency of sign and interpretation.

Moderate asymmetries are expected in human movement, but values exceeding ±10–15% are commonly regarded as clinically relevant. High LSI values may reflect unilateral weakness, incomplete rehabilitation, or compensatory strategies that can increase injury risk.

Co-Contraction Index (CCI, Early Stance)—The CCI quantifies the degree of simultaneous activation of hamstrings (H) and quadriceps (Q), reflecting how much the two

muscle groups co-activate to stabilize the knee during loading. It is computed at each instant of time and then averaged over early stance:

$$\mathrm{CCI}(t) = \frac{2 \cdot \min\backslash big\big(A_H(t), A_Q(t)\backslash big\big)}{A_H(t) + A_Q(t)}, \quad \mathrm{CCI_{ES}} = \frac{1}{T_{ES}} \int_{\text{early stance}} \mathrm{CCI}(t)\ dt$$

where $A_H(t)$ and $A_Q(t)$ denote the time-varying normalized activations (or synthetic force proxies) of hamstrings and quadriceps. The numerator takes the smaller activation at each time point, ensuring that only overlapping activity is counted, while the denominator normalizes by the total activation of both muscle groups. As a result, CCI(t) ranges between 0 (no overlap) and 1 (perfect co-activation). The second formula computes the average CCI across the duration of early stance, where T_{ES} is the time length of that phase. Synthetic activations were normalized to a 0–1 scale before computation, and the index was averaged across the first 25% of stance to reflect early-loading stabilization.

A moderate level of co-contraction is beneficial for knee stability, especially after foot-strike when external loads are high. However, excessively high CCI values may indicate inefficient movement strategies, increased joint compression, and reduced energy efficiency, whereas very low values may compromise joint stability.

In addition to $H{:}Q_{dyn}$, LSI, and CCI, supplementary features were derived to capture temporal and external load characteristics. These included the time to peak knee flexion moment (TTP-KFM), reflecting the timing of flexor demand relative to stance, and the vertical ground reaction force limb symmetry index (vGRF LSI), which quantified external load asymmetry between limbs. Both indices provided complementary information on joint loading strategies and neuromechanical control.

Time-to-Peak Knee Flexion Moment (TTP-KFM)—This feature represents the temporal coordination of knee joint loading. It is defined as the percentage of the gait cycle from foot-strike to the occurrence of maximum knee flexion moment:

$$TTP_{KFM} = 100 \cdot \frac{t_{peak} - t_{FS}}{t_{cycle}} \backslash\%$$

where t_{peak} is the time of maximum knee flexion moment, t_{FS} is the foot-strike event, and t_{cycle} is the full gait cycle duration. The result is expressed as a percentage of the cycle. Foot-strike and toe-off were detected from vertical GRF > 20 N, and time-to-peak was expressed as a percentage of stance duration.

A delayed or premature time-to-peak may reflect altered neuromuscular strategies, compensatory patterns after injury, or fatigue-induced timing shifts.

Vertical GRF Peak LSI—Ground reaction force (GRF) symmetry reflects external loading balance between limbs. It was quantified using the same LSI formula as for joint moments:

$$\mathrm{LSI_{vGRF}} = 100 \cdot \frac{F_{dom} - F_{non-dom}}{0.5 \cdot (F_{dom} + F_{non-dom})} \backslash\%$$

where F_{dom} and $F_{non\text{-}dom}$ are the peak vertical GRFs measured (or simulated) for the dominant and non-dominant limb.

Higher asymmetry in vGRF peaks can indicate unilateral weakness, residual deficits after injury, or compensatory strategies that shift loading away from the weaker limb.

Stride-to-stride irregularities were quantified using the coefficient of variation (CV), applied to selected biomechanical outputs. Low CV values indicated stable and consistent performance, whereas higher CV reflected instability, compensatory adjustments, or neuromuscular fatigue. This ensured that the feature set remained sensitive to both static thresholds and dynamic variability.

Variability Metrics (Coefficient of Variation, CV)—Stride-to-stride variability is a measure of movement consistency. Increased variability often reflects instability, fatigue, or insufficient neuromuscular control. The coefficient of variation was applied to selected features such as dynamic H:Q ratios and LSI indices:

$$CV = 100 \cdot \frac{\sigma}{\mu} \backslash\%$$

where σ is the standard deviation and μ is the mean of the feature across multiple strides. The result indicates relative variability as a percentage.

Low CV values indicate consistent motor patterns and good control, while high CV values suggest instability, irregular loading, or compensatory strategies.

Finally, running speed was included as a contextual covariate to account for velocity-dependent differences in kinetics and coordination. Although not a clinical indicator per se, speed provided an essential control variable to ensure comparability across trials and conditions.

Contextual Variable (Running Speed)—Running speed (vvv, in $m \cdot s^{-1}$) was included as a continuous covariate. Speed is known to strongly affect joint moments, GRFs, and timing variables. By including speed as a predictor, we controlled for velocity-dependent differences in biomechanics:

$$v = \frac{d}{t}$$

where d is the running distance and t is the time.

Including running speed as a contextual covariate allowed us to account for velocity-dependent changes in kinetics and coordination, ensuring that the derived features were comparable across trials performed at different intensities.

Taken together, the feature set captured complementary aspects of hamstrings–quadriceps balance, limb symmetry, co-contraction, and variability, ensuring that subsequent model predictions remained anchored in biomechanical constructs rather than abstract numerical patterns. To facilitate clarity and reproducibility, all simulation-derived biomechanical features are summarized in Table S2, together with their defining equations, labeling thresholds, and biomechanical interpretations. These features were then used as inputs for model training and validation, forming the next stage of the framework.

Table S2. Overview of simulation-derived biomechanical features.

Feature	Simplified formula	Captures	Thresholds used in labeling	Clinical relevance	Interpretation when elevated/deviant
**Dynamic H:Q ratio**	$\int M_{flex} / \int M_{ext}$	Balance of hamstrings vs. quadriceps effort	< 0.60 or > 1.20	Central for knee stability; relates to ACL strain and hamstring overuse	Low = quadriceps dominance (↑ ACL risk);

					High = hamstring overcompensation
**Limb Symmetry Index (LSI)**	$(X_{dom}-X_{non\text{-}dom})/(0.5 (X_{dom}+X_{non\text{-}dom}))\times100\%$	Inter-limb asymmetry (moments, stance, vGRF)	> ±12% (for knee moment LSI)	Widely used in rehabilitation and return-to-sport	High values = unilateral weakness, incomplete recovery
**Co-Contraction Index (CCI)**	$2\cdot\min(A_H,A_Q)/(A_H+A_Q)$ averaged over stance	Degree of hamstrings–quadriceps overlap	> 0.58	Reflects neuromuscular stabilization strategy	Moderate = stability; High = inefficiency, joint compression
**Time-to-Peak Knee Flexion Moment**	$(t_{peak}-t_{FS})/t_{cycle}\times100\%$	Temporal coordination of knee loading	Not used as threshold	Sensitive to compensations, fatigue, injury recovery	Deviations = altered coordination, compensatory strategy
**Vertical GRF Peak LSI**	$(X_{dom}-X_{non\text{-}dom})/(0.5 (X_{dom}+X_{non\text{-}dom}))\times100\%$	External load symmetry	Not used as threshold	Indicator of load distribution across limbs	High = unloading weaker limb, residual deficits
**Variability (CV)**	$\sigma/\mu\times100\%$	Stride-to-stride consistency	Not used as threshold	Proxy for stability, fatigue, motor control	High = instability/fatigue; Low = stable control
**Running speed**	$v = d / t$	Contextual covariate	Controlled covariate	Essential to normalize biomechanical comparisons	Faster speeds = higher joint loads, altered timing

Notes: Dynamic H:Q ratio and CCI are dimensionless indices; LSI, variability (CV), and time-to-peak KFM are expressed as percentages; running speed is in $m\cdot s^{-1}$.

The summary provided in Table S2 highlights how each simulation-derived feature was explicitly grounded in biomechanical theory and linked to clinically recognized thresholds.

By combining indices of muscle balance, inter-limb symmetry, co-contraction, temporal coordination, and variability, the framework captures complementary aspects of knee joint function. This integration ensures that model predictions are not abstract outputs but remain directly interpretable within established clinical paradigms of injury risk, rehabilitation, and return-to-sport decision-making. In this way, the feature set constitutes a physiologically meaningful foundation for the subsequent machine learning analyses.

S1.3 Model Training and Validation

S1.3.1. Computational Model and Data Generator

The synthetic data generator was implemented as a computational module designed to instantiate virtual subjects, simulate running trials, embed variability across speeds and strides, and assign class labels based on biomechanical thresholds. This modular pipeline served as the technical engine of the framework, translating methodological assumptions into reproducible digital data streams.

Computational Model—The synthetic data generator was implemented as a computational module designed to instantiate virtual subjects, simulate running trials with controlled variability across speeds and strides, and assign class labels based on biomechanical thresholds. This modular pipeline served as the technical engine of the framework, translating methodological assumptions into reproducible digital data streams.

The computational framework was designed to emulate the principal outputs of musculoskeletal simulations without implementing a full forward-dynamics solver. Rather than estimating forces and moments from experimental kinematics, a reduced but physiologically meaningful model was constructed to generate synthetic quantities aligned with established biomechanical concepts. Hamstrings–quadriceps interaction was

operationalized dynamically through flexor–extensor moment balance, co-contraction indices, and symmetry metrics. This approach ensured full control of variability, transparent labeling of imbalance conditions, and reproducibility of methodological steps.

A summary of the model components, their implementation, and rationale is provided in Table S3, consolidating the computational assumptions that guided the generation of synthetic data.

Table S3. Computational model assumptions, implementation details, and their methodological impact.

Aspect	Implementation in model	Rationale	References / common use	Impact on labeling	Expected effect on ML performance	Interpretability dimension
**Population heterogeneity**	160 virtual subjects; latent imbalance propensity from bimodal Gaussian	Mimics variation in athletes; ensures balanced/imbalanced clusters	Simulation-based heterogeneity	Indirect	Provides realistic variance for model generalization	Contextual variability
**Flexor–extensor balance**	Dynamic H:Q ratio (integrated moments)	Central to ACL stability, hamstring injury risk	Gold-standard ratio in sports medicine	Direct (H:Q <0.60 or >1.20)	Major predictor of imbalance	Muscle balance
**Inter-limb asymmetry**	LSIs for knee moment, stance, vGRF	Reflects unilateral weakness or compensatory loading	Return-to-sport criterion (±10–15%)	Direct (knee moment LSI >12%)	Strong discriminative power	Symmetry / load distribution
**Neuromuscular control**	Synthetic activations → CCI	Captures stabilizing co-contraction	EMG/simulation practice	Direct (CCI >0.58)	Moderate predictor; adds nuance	Stability strategy
**Temporal coordination**	Time-to-peak knee flexion moment	Identifies compensatory timing shifts	Fatigue/post-injury gait analyses	None	Secondary role; refines interpretation	Timing / coordination
**Variability modeling**	Gaussian noise; CV computed	Mimics stride-to-stride inconsistency	Variability as control proxy	None	Adds robustness; improves error analysis	Motor consistency
**Measurement noise**	~5% random label flips	Emulates misclassification and imperfect ground truth	Standard ML validation	Indirect	Stress test for classifier calibration	Label uncertainty
**Label definition**	Composite thresholds (H:Q, LSI, CCI)	Anchors imbalance in task-specific biomechanical rules	Sports medicine thresholds	Direct	Ensures ground-truth plausibility	Clinical face validity

Notes: The computational framework emulated musculoskeletal simulation outputs without implementing a full solver, and thresholds were selected for methodological demonstration rather than clinical cut-off values.

As shown in Table S3, the computational set-up not only specifies how synthetic features were generated, but also clarifies their role in class labeling, expected influence on classification performance, and interpretability dimension. This level of transparency ensures that methodological decisions are explicitly linked to biomechanical constructs rather than treated as abstract modeling choices.

In summary, the computational model provided a controlled and physiologically grounded framework in which imbalance could be represented transparently through a small set of interpretable variables. By combining biomechanical plausibility with methodological flexibility, the set-up established a reproducible foundation on which subsequent stages of data generation and analysis were built.

Synthetic Data Generation—Synthetic running trials were generated to emulate the biomechanical outputs typically obtained from musculoskeletal simulations while retaining full control over variability, balance between classes, and reproducibility. A virtual cohort of 160 subjects was created, each contributing between two and five trials across

three speed conditions representative of natural locomotor demands: slow (~2.8 m·s^{-1}), moderate (~3.4 m·s^{-1}), and fast (~4.2 m·s^{-1}). In total, 573 trials were produced, providing sufficient heterogeneity for model training and evaluation.

To embed inter-individual variation, each subject was assigned a latent *imbalance propensity* parameter sampled from a bimodal Gaussian distribution. This construct reflected the dichotomy between balanced and imbalanced neuromechanical profiles commonly observed in athletic populations. From these latent values, synthetic biomechanical features were derived, including dynamic H:Q ratio, knee moment LSI, stance-time LSI, vertical GRF LSI, co-contraction index (CCI), and time-to-peak knee flexion moment. Gaussian noise was added to each feature to approximate both measurement error and stride-to-stride variability, ensuring stochastic variability consistent with empirical datasets.

Class labels were assigned using a composite rule grounded in biomechanical plausibility. A trial was classified as *imbalanced* if any of the following conditions were exceeded: dynamic H:Q ratio < 0.60 or > 1.20; |knee moment LSI| > 12%; or early-stance CCI > 0.58. These thresholds align with values reported in sports medicine, where H:Q cut-offs are frequently debated as indicators of muscle imbalance, LSI deviations above 10–15% are considered clinically meaningful, and elevated co-contraction indices have been associated with compensatory or inefficient stabilization strategies. To mimic the imperfect ground truth typical of real assessments, ~5% of labels were stochastically flipped. This deliberate injection of label noise acted as a stress test for classifier robustness and prevented overfitting to idealized conditions.

The composite rules used to assign imbalance labels are summarized in Table S4, which consolidates the thresholds, biomechanical rationale, and methodological impact of each criterion.

Table S4. Composite criteria for imbalance labeling, their biomechanical basis, and methodological implications.

Criterion	Threshold	Biomechanical relevance	Expected biomechanical consequence	Interpretability dimension	Evidence base	Role in model evaluation
**Dynamic H:Q ratio**	< 0.60 or > 1.20	Proxy for hamstrings–quadriceps balance; debated cut-offs in sports medicine	Low ratio → ↑ ACL strain; High ratio → hamstring overuse	Muscle balance	Commonly reported in isokinetic & simulation studies	Used for labeling and as top predictor in ML
**Knee moment LSI**	> 12% (absolute)	Indicator of inter-limb asymmetry in joint loading	Reflects unilateral weakness or compensatory strategies	Symmetry / load distribution	±10–15% widely used as return-to-sport cut-off	Labeling criterion and interpretable asymmetry index
**Early-stance CCI**	> 0.58	Quantifies stabilizing co-activation of hamstrings and quadriceps	Excessive co-contraction → ↑ joint compression, inefficient stabilization	Stability strategy	Documented in EMG and simulation contexts	Labeling criterion and interpretability dimension
**Random label noise**	~ 5%	Mimics imperfect ground truth and misclassification	Increases robustness to uncertainty	Label uncertainty	Standard ML stress-test technique	Labeling only (not used as predictor)

Notes: Criteria marked by explicit thresholds (H:Q ratio, knee moment LSI, CCI) contributed directly to class labeling, while random label noise acted indirectly by simulating imperfect ground truth.

By integrating latent subject parameters, stochastic perturbations, and clinically grounded thresholds, the synthetic dataset established a reproducible and physiologically coherent environment for evaluating the proposed framework. Beyond methodological demonstration, this design serves as a translational blueprint, ensuring that insights

derived from simulation can be extended and validated on empirical running datasets in applied sports medicine contexts.

On this computational basis, the next step involved configuring and validating machine learning classifiers trained on the generated biomechanical features.

S1.3.2 Machine Learning Configuration, Validation Strategy, and Interpretability Procedures.

The machine learning component of the framework was designed to combine predictive accuracy with methodological transparency. Gradient Boosting was selected as the primary classifier given its balance of flexibility and interpretability, while a calibrated logistic regression served as a linear baseline for benchmarking. Probability estimates were calibrated using isotonic regression, ensuring that outputs could be interpreted as meaningful risk estimates rather than arbitrary scores. Validation was carried out at the subject level using a GroupKFold design, preventing data leakage across trials and ensuring independence between training and evaluation.

The classification framework was implemented using Gradient Boosting, a decision tree–based ensemble algorithm that constructs models sequentially to minimize prediction error. Gradient Boosting was selected because it offers a balance between predictive performance and interpretability: unlike deep neural networks or other black-box models, its structure allows for transparent feature importance analyses and partial dependence visualizations. As a benchmark, a calibrated logistic regression model was also implemented to provide a linear baseline against which to contrast the performance of the non-linear ensemble.

To reduce the risk of overfitting while retaining sufficient flexibility, the base learners were restricted to shallow decision trees with a maximum depth of three. Shallow trees constrain the number of interaction terms captured by each base learner, ensuring that the resulting ensemble remains both stable and interpretable. This choice reflects a deliberate compromise: deeper trees could increase raw predictive accuracy at the expense of interpretability, whereas overly shallow learners (depth one or two) risk discarding meaningful biomechanical interactions.

Probability estimates were calibrated using isotonic regression to ensure that model outputs represented meaningful likelihoods of imbalance rather than arbitrary scores. This method was preferred over Platt scaling because it does not assume linearity in the log-odds space and is better suited for non-linear decision boundaries. Alternative calibration methods such as temperature scaling or Bayesian binning were also considered, but isotonic regression was selected for its non-parametric flexibility and superior probability calibration performance in ensemble models. Calibrated probabilities enable direct clinical interpretation, linking predicted risk with the empirical likelihood of exceeding imbalance thresholds. Explicit reporting of calibration slope and intercept reinforces this interpretability by quantifying the agreement between predicted probabilities and observed outcome frequencies. This choice aligns with prior evidence that isotonic regression yields better-calibrated probabilities than Platt scaling across a wide range of non-linear classifiers.

Validation followed a subject-wise GroupKFold strategy with k = 5. All trials belonging to a given virtual subject were confined to the same fold, preventing information leakage between training and testing sets. Each fold contained approximately 32 subjects (≈115 trials), ensuring balanced representation of both balanced and imbalanced profiles and full subject-level independence between partitions. This design choice is essential in biomechanics, where trial-level leakage can artificially inflate performance metrics if trials from the same subject appear in both training and testing partitions. Subject-wise grouping ensures that the reported performance reflects generalization to unseen individuals rather than repeated measurements of the same entity.

Model performance was assessed across multiple complementary metrics: ROC-AUC (discrimination across thresholds), PR-AUC (precision–recall trade-off under class imbalance), balanced accuracy (robustness to uneven class prevalence), F1 score (harmonic mean of precision and recall), and Brier score (calibration of probabilistic predictions). To quantify statistical uncertainty, 2000× bootstrap resampling was applied to out-of-fold predictions, yielding confidence intervals for each metric. Bootstrap confidence intervals were computed by resampling subjects with replacement and retaining all their trials within each replicate, thereby preserving within-subject dependence. This cluster-level bootstrap design ensured that uncertainty estimates were not artificially narrowed by treating repeated trials as independent. Bootstrap resampling is a widely recommended method for estimating sampling variability and provides robust confidence intervals on cross-validated predictions. The number of bootstrap iterations was chosen to provide stable estimates while avoiding computational inefficiency, following recommendations from prior methodological studies. No classical hypothesis testing (e.g., p-values, effect sizes) was performed, as the dataset is fully synthetic; instead, bootstrap confidence intervals on cross-validated predictions provide the statistical quantification of robustness.

Interpretability was approached at both the global and local levels. Permutation feature importance quantified global predictor relevance, while partial dependence plots (PDPs) visualized the marginal effects of each biomechanical variable on imbalance probability, providing complementary global and local interpretability. Globally, permutation feature importance was calculated on a hold-out set to identify the predictors that contributed most strongly to classification performance, with this method preferred over impurity-based scores due to its robustness against feature correlation. Locally, partial dependence plots were generated to visualize the marginal effect of individual predictors on predicted imbalance risk. This dual strategy ensured that classification decisions could be traced back to explicit biomechanical constructs such as flexor–extensor balance, asymmetry, and co-contraction, rather than remaining opaque algorithmic outputs. To complement these analyses, calibration slope and intercept were inspected to evaluate the reliability of probability estimates, while decision-curve analysis quantified net benefit across a range of thresholds. Taken together, these procedures strengthened the transparency of the modeling pipeline and reinforced the physiological plausibility of the predictions.

Reporting of the modeling pipeline follows the TRIPOD-AI recommendations for transparency in machine learning-based prediction models.

An overview of the model parameters, validation scheme, and evaluation metrics is summarized in Table S5.

Table S5. Machine learning model configuration, validation strategy, and evaluation procedures.

Component	Implementation	Rationale	Impact on analysis	Interpretability dimension
**Algorithm**	Gradient Boosting, decision tree base learners	Balances predictive accuracy with transparency; widely used in biomedical and sports data	Provides non-linear modeling without black-box opacity	Allows feature importance and partial dependence analysis
**Tree depth**	max_depth = 3	Prevents overfitting; restricts complexity to meaningful biomechanical interactions	Ensures stable generalization across subjects	Shallow trees preserve clarity of marginal effects
**Probability calibration**	Isotonic regression	Produces calibrated risk estimates; superior to Platt scaling for non-linear boundaries	Probabilities can be interpreted as empirical imbalance risk	Enhances clinical interpretability of outputs
**Validation scheme**	Subject-wise GroupKFold, k = 5	Prevents data leakage; ensures subject-independent evaluation	Metrics reflect generalization to unseen individuals	Strengthens methodological rigor
**Baseline model**	Logistic regression (calibrated)	Provides linear reference for benchmarking GBM	Demonstrates added value of non-linear modeling	Coefficients interpretable as linear effects
**Performance metrics**	ROC-AUC, PR-AUC, balanced accuracy, F1, Brier score	Capture discrimination, calibration, and robustness	Multi-dimensional evaluation of classifier	Supports transparent reporting
**Uncertainty quantification**	2000× bootstrap on out-of-fold predictions	Estimates confidence intervals for all metrics	Demonstrates robustness of findings	CI reporting aids reproducibility
**Global interpretability**	Permutation feature importance (hold-out set)	Identifies predictors most influential for classification	Links model output to biomechanical determinants	Highlights task-specific predictors (H:Q, LSI, CCI)
**Local interpretability**	Partial dependence plots	Visualizes marginal predictor effects	Ensures plausibility of model decisions	Direct mapping to biomechanical constructs

Notes: The table provides a schematic overview of the machine learning pipeline, serving as a concise reference for algorithm configuration, validation, and evaluation.

As consolidated in Table S5, the configuration of the machine learning pipeline reflects a deliberate balance between predictive robustness and biomechanical interpretability. Each component — from algorithm choice and calibration to validation design and interpretability methods — was selected to minimize bias, prevent information leakage, and ensure that the model's outputs could be transparently linked to underlying physiological constructs. This alignment of technical rigor with biomechanical meaning established a methodological foundation suitable for reproducible evaluation in the subsequent analyses.

Together, these configuration and validation procedures provided a rigorous foundation for the subsequent evaluation of robustness, optimization, interpretability, and bias.

S1.3.3. Rule-Based Baseline (Deterministic Classifier Derived from the Label Definition)

Rationale—To provide a transparent benchmark and to quantify the added value of machine learning beyond the composite clinical rule used for labeling, we implemented a rule-based baseline that exactly mirrors the study's imbalance definition.

Deterministic decision rule—A trial was classified as "imbalanced" if any of the following conditions held: (i) dynamic hamstrings–to–quadriceps ratio (H:Qdyn) < 0.60 or > 1.20; or (ii) absolute knee-moment limb symmetry index (|LSI|) > 12%; or (iii) early-stance

co-contraction index (CCI) > 0.58. Otherwise, the trial was classified as "balanced". This rule is identical to the composite thresholding used to assign labels in the synthetic cohort.

Continuous rule score (for ROC/PR/DCA). To enable ranking and probability-based evaluation, we derived a monotone, continuous rule severity score (s_rule) from distance-to-threshold:

z_HQ = max[(0.60 – H:Qdyn)/0.60, (H:Qdyn – 1.20)/1.20, 0]

z_LSI = max[|LSI| – 0.12, 0] / 0.12

z_CCI = max[CCI – 0.58, 0] / 0.58

s_rule = max(z_HQ, z_LSI, z_CCI)

The raw score was s_rule = max(z_HQ, z_LSI, z_CCI), with s_rule = 0 indicating no threshold exceedance. For comparability across folds, s_rule was min–max scaled within the training data of each fold and then passed through isotonic regression to produce calibrated probabilities.

Evaluation protocol—The rule-based classifier was evaluated under the same subject-wise GroupKFold design (k = 5) used for the machine-learning models to guarantee subject-independent estimates. Performance metrics included ROC-AUC, PR-AUC, balanced accuracy, F1 score, and Brier score; 2,000× bootstrap resampling of out-of-fold predictions provided 95% confidence intervals. For probability-based variants (continuous s_rule), isotonic calibration was fit strictly on the training partitions within each fold and applied to the corresponding test partitions to avoid leakage. Decision-curve analysis compared net benefit across probability thresholds against "treat-all" and "treat-none" references.

Reporting—We report the deterministic rule performance (binary predictions), and the continuous, calibrated rule score, which preserves the rule's interpretability while enabling ranking in ROC/PR and threshold-based clinical utility in DCA.

S1.3.4. Evaluation and Transparency

Beyond configuration and validation, a rigorous evaluation was required to establish both the methodological reliability and the physiological plausibility of the framework. This evaluation extended in four complementary directions: sensitivity and robustness analyses tested the stability of imbalance definitions under varied thresholds and noise; hyperparameter optimization assessed the consistency of model performance across different configurations; interpretability procedures linked predictions back to muscular constructs, ensuring that outputs could be understood in biomechanical terms; and risk-of-bias analyses exposed the limitations inherent to in silico simplifications. Together, these components provided a comprehensive perspective on the trustworthiness of the digital assessment approach.

Sensitivity and Robustness Analyses—Robustness of the proposed framework was evaluated through a structured series of sensitivity analyses targeting the most influential methodological choices: label thresholds, noise levels, and cross-validation stability. These perturbations were not arbitrary but corresponded to physiologically relevant muscular constructs: dynamic H:Q cut-offs mirror debated definitions of hamstring–quadriceps balance, knee moment LSI thresholds align with clinical return-to-sport criteria for quadriceps and hamstrings, and co-contraction thresholds reflect compensatory strategies of simultaneous flexor–extensor activation. By anchoring robustness tests in muscular imbalance definitions, the evaluation ensured that methodological stability was interpreted in a biomechanically meaningful context.

First, imbalance thresholds were perturbed within ranges grounded in sports medicine practice. For the dynamic H:Q ratio, cut-offs were shifted from 0.55 to 0.65 on the lower end and from 1.15 to 1.25 on the upper end, reflecting ongoing debates about whether universal cut-offs are too strict or too lenient. Similarly, the knee moment LSI threshold was varied between 10%, 12%, and 15%, values commonly used in return-to-sport assessments. For co-contraction index (CCI), the critical value was adjusted between 0.55 and 0.60, spanning both conservative and liberal definitions of inefficient stabilization.

Second, robustness was tested against random label noise, designed to mimic imperfect ground truth. Beyond the default 5%, scenarios with 0% noise (idealized labels) and 10% noise (stress test) were examined to evaluate how much performance was affected when misclassification increased.

Third, cross-validation replicability was tested by repeating subject-wise GroupKFold splits across five different random seeds. Stability of bootstrap confidence intervals across replications was considered evidence of robustness.

Finally, evaluation went beyond discrimination alone, incorporating additional indicators recommended in current reporting guidelines.. We quantified class prevalence shifts caused by changing thresholds, verified calibration slopes remained close to unity, and estimated net benefit at a decision threshold of 0.5 to assess potential clinical utility. Confidence interval widths were explicitly reported as a measure of statistical uncertainty, while performance differences relative to both the baseline model and a calibrated logistic regression were computed to contextualize results.

Overall, results confirmed that the framework was not contingent on a single labeling choice or random seed. Even under the strictest perturbations (H:Q shifted ±0.05, LSI raised to 15%, CCI raised to 0.60, or 10% random noise), ROC-AUC remained above 0.91, balanced accuracy above 0.91, and F1 scores above 0.92. Calibration slopes stayed within 0.93–1.02, while decision-curve net benefit remained consistently positive. Importantly, improvements over logistic regression (ΔROC ≈ +0.21 to +0.24) were preserved across all conditions. Net Benefit was estimated using standard decision-curve analysis methodology, providing a measure of potential clinical utility across threshold scenarios. Performance values above 0.90 for ROC-AUC and above 0.93 for balanced accuracy are generally interpreted as excellent in biomechanical and clinical modeling contexts, confirming that these results represent high predictive reliability.

The full set of results is summarized in Table S6, which integrates discrimination, calibration, uncertainty quantification, prevalence, and clinical utility indicators into a comprehensive overview.

Table S6. Sensitivity and robustness analyses across varying labeling thresholds, noise levels, and validation seeds.

Condition	ROC-AUC (95% CI)	CI Width	PR-AUC (95% CI)	Balanced Acc. (95% CI)	F1 (95% CI)	Brier	Calib. slope	Class prevalence (% imbalanced)	Net Benefit @0.5	ΔROC vs. baseline	ΔROC vs. Logistic
**Baseline (H:Q <0.60/>1.20; LSI >12%; CCI >0.58; 5% noise)**	0.933 (0.908–0.958)	0.050	0.918 (0.892–0.943)	0.943 (0.924–0.962)	0.940 (0.919–0.958)	0.056	1.00	47.3%	0.34	–	+0.230

**H:Q thresholds 0.55 / 1.25**	0.927 (0.900–0.952)	0.052	0.912 (0.886–0.939)	0.936 (0.916–0.955)	0.934 (0.913–0.952)	0.060	0.98	45.1%	0.33	–0.006	+0.224
**H:Q thresholds 0.65 / 1.15**	0.921 (0.892–0.948)	0.056	0.907 (0.879–0.934)	0.932 (0.910–0.951)	0.928 (0.905–0.947)	0.064	0.97	49.6%	0.32	–0.012	+0.218
**LSI threshold 10%**	0.936 (0.911–0.959)	0.048	0.920 (0.895–0.945)	0.946 (0.926–0.963)	0.942 (0.921–0.959)	0.055	1.01	52.1%	0.35	+0.003	+0.233
**LSI threshold 15%**	0.922 (0.896–0.948)	0.052	0.909 (0.882–0.936)	0.931 (0.909–0.950)	0.927 (0.904–0.946)	0.066	0.96	43.7%	0.31	–0.011	+0.219
**CCI threshold 0.55**	0.934 (0.910–0.958)	0.048	0.919 (0.893–0.944)	0.944 (0.925–0.962)	0.940 (0.918–0.958)	0.057	1.00	48.5%	0.34	+0.001	+0.231
**CCI threshold 0.60**	0.918 (0.889–0.945)	0.056	0.905 (0.877–0.932)	0.929 (0.907–0.949)	0.925 (0.902–0.944)	0.068	0.95	46.8%	0.30	–0.015	+0.214
**Noise 0% (ideal labels)**	0.939 (0.915–0.961)	0.046	0.923 (0.898–0.947)	0.948 (0.929–0.964)	0.945 (0.924–0.962)	0.052	1.02	47.0%	0.35	+0.006	+0.236
**Noise 10% (stress test)**	0.910 (0.880–0.939)	0.059	0.893 (0.864–0.922)	0.917 (0.894–0.938)	0.919 (0.895–0.940)	0.073	0.93	47.6%	0.29	–0.023	+0.207

Notes. Metrics are reported as mean values with 95% bootstrap CIs. The consistency of performance across all tested scenarios confirms that the framework is stable and resilient, reinforcing its value as a methodological baseline for future validation.

To ensure full transparency and reproducibility, each metric reported in Table S6 is explicitly defined and contextualized as follows: ROC-AUC refers to the area under the Receiver Operating Characteristic curve, where values above 0.90 are typically interpreted as excellent discrimination. Confidence intervals (CI) were derived using the same bootstrap procedure, while the CI width represents the difference between upper and lower bounds and is reported as an indicator of statistical uncertainty. PR-AUC denotes the area under the Precision–Recall curve, which is particularly informative when class distributions are not perfectly balanced. Balanced accuracy is calculated as the mean of sensitivity and specificity, thereby correcting for unequal class prevalence. The F1 score represents the harmonic mean of precision and recall and reflects the trade-off between false positives and false negatives.

The Brier score quantifies the mean squared error of probabilistic predictions, with lower values indicating better calibration. Calibration slope corresponds to the regression slope between predicted probabilities and observed outcomes, with values close to 1.0 indicating well-calibrated estimates. Class prevalence indicates the proportion of trials labeled as imbalanced under each threshold scenario, thus making explicit how definitional choices affect the dataset distribution. Net benefit was estimated at a probability threshold of 0.5 following standard decision-curve analysis methodology, where positive values denote clinical utility compared to default strategies.

Finally, ΔROC vs. baseline reflects the absolute difference in ROC-AUC relative to the primary configuration (H:Q <0.60/>1.20, LSI >12%, CCI >0.58, with 5% label noise), while ΔROC vs. Logistic quantifies the gain over the calibrated logistic regression benchmark used as a linear reference model. It should be emphasized that all thresholds and noise levels were chosen for methodological demonstration rather than as clinical cut-off values.

Sensitivity to threshold definitions—Performance remained consistently high even when imbalance definitions were perturbed within clinically plausible ranges. Shifting H:Qdyn_{dyn}dyn cut-offs from 0.60/1.20 to 0.55/1.25 or 0.65/1.15 yielded AUROC values of ≈0.92–0.93 and balanced accuracy of ≈0.93–0.94. Similarly, varying the knee moment LSI threshold between 10% and 15% produced AUROC values of ≈0.92–0.94 and balanced accuracy of ≈0.93–0.95. These adjustments altered class prevalence by only ≈2–3%, confirming that model discrimination and calibration are not artifacts of a single numerical cut-off but persist across clinically recognized ranges. This robustness supports H1 and underscores that the framework's utility derives from consistent biomechanical signal rather than dependence on an exact threshold value.

Overall, these robustness analyses confirm that hamstring–quadriceps imbalance can be consistently detected across varied thresholds, noise levels, and running speeds. The persistence of high discrimination and calibration under such perturbations reinforces the muscular validity of the framework, showing that detection is not contingent on arbitrary definitions but reflects fundamental neuromuscular patterns. To confirm that the simulated feature ranges reflect physiological realism, we compared the distributions of H:Qdyn, LSI, and CCI generated by the framework with values reported in empirical biomechanics literature. The simulated dynamic H:Q ratios (≈0.6–1.2), knee moment LSI values (≈±10–15%), and early-stance co-contraction indices (≈0.5–0.6) are consistent with experimental findings from isokinetic and IMU-based assessments of running and rehabilitation tasks [9,12,37,38]. This alignment supports that the in-silico generator reproduces realistic neuromuscular variability rather than arbitrary numerical ranges, reinforcing the external plausibility of the framework.

Hyperparameter Optimization—The hyperparameter optimization process was grounded in muscle-relevant features, ensuring that model parameters preserved the interpretability of hamstring–quadriceps imbalance detection. Although hyperparameter optimization is a computational process, its stability is critical for ensuring that muscular interpretations remain reliable. Consistent classification across parameter ranges confirms that detection of hamstring–quadriceps imbalance emerges from physiological features (H:Q ratio, limb symmetry indices, co-contraction) rather than from fragile model tuning.

The Gradient Boosting classifier was configured through a structured hyperparameter optimization process to ensure robust performance without overfitting. A grid search explored combinations of maximum tree depth (2–5), number of estimators (50–500), and learning rate (0.01–0.20), while keeping subsample ratios and feature sampling at default values for transparency. Early stopping on out-of-fold predictions was applied to prevent excessive iterations when performance plateaued.

Each hyperparameter configuration was evaluated under the same subject-wise GroupKFold strategy with isotonic calibration as in the primary analysis. Performance was assessed using ROC-AUC, PR-AUC, balanced accuracy, F1, Brier score, and calibration slope. In addition, ΔROC relative to the baseline configuration and ΔROC vs. logistic regression were computed to contextualize model improvements. To quantify uncertainty, bootstrap resampling was applied to each configuration, and confidence interval widths were reported.

Results confirmed that performance was relatively insensitive to moderate changes in learning rate and number of estimators, provided that tree depth remained between 3 and 4. Very shallow trees (depth = 2) slightly underfit, while deeper trees (depth = 5) provided marginal ROC-AUC gains but increased variance and calibration error. The selected configuration (depth = 3, learning rate = 0.10, 200 estimators) achieved the optimal trade-off between discrimination, calibration, and stability. Key results of the hyperparameter optimization are summarized in Table S7, showing how variations in tree depth, number of estimators, and learning rate influenced discrimination, calibration, and overall stability.

Table S7. Hyperparameter optimization results

Depth	Estimators	Learning Rate	ROC-AUC (95% CI)	CI Width	PR-AUC	Balanced Acc.	F1	Brier	Calib. slope	ΔROC vs. baseline	ΔROC vs. Logistic
**2**	100	0.10	0.902 (0.873–0.931)	0.058	0.881	0.906	0.908	0.082	0.91	–0.031	+0.199
**2**	200	0.10	0.910 (0.882–0.938)	0.056	0.889	0.913	0.916	0.078	0.92	–0.023	+0.207
**3**	100	0.05	0.928 (0.902–0.953)	0.051	0.909	0.935	0.932	0.062	0.98	–0.005	+0.225
**3**	200	0.10 (final)	0.933 (0.908–0.958)	0.050	0.918	0.943	0.940	0.056	1.00	–	+0.230
**3**	500	0.10	0.936 (0.910–0.960)	0.050	0.920	0.946	0.943	0.054	0.99	+0.003	+0.233
**4**	200	0.10	0.939 (0.913–0.962)	0.049	0.922	0.947	0.944	0.052	1.02	+0.006	+0.236
**5**	200	0.10	0.941 (0.914–0.965)	0.051	0.924	0.949	0.946	0.051	1.08	+0.008	+0.238
**3**	200	0.20	0.927 (0.900–0.953)	0.053	0.907	0.934	0.930	0.061	1.04	–0.006	+0.224

Notes. Metrics are reported as mean values with 95% bootstrap CIs. Hyperparameter configurations were systematically explored, and results confirmed that performance remained stable across a wide range of settings. The selected configuration balanced discrimination, calibration, and efficiency, providing a transparent and reproducible foundation for subsequent analyses.

The analysis demonstrated that model performance was relatively insensitive to moderate changes in learning rate and number of estimators, provided that tree depth remained within 3–4. Shallow trees (depth = 2) consistently underfit, yielding lower discrimination and calibration slope values. Deeper trees (depth = 5) produced marginal gains in ROC-AUC (≈ +0.008) but at the expense of calibration stability (slope > 1.05), suggesting overfitting. The configuration with depth = 3, learning rate = 0.10, and 200 estimators was therefore retained as the final model, offering the optimal balance between discrimination (ROC-AUC = 0.933), calibration (slope ≈ 1.00), and computational efficiency. This selected setting served as the reference point for subsequent analyses. Taken together, these results indicate that robust detection of hamstring–quadriceps imbalance does not depend on finely tuned hyperparameter settings, but rather on the consistent physiological signal captured by dynamic H:Q ratios, symmetry indices, and co-contraction features.

Risk of Bias and Assumptions—Given the in silico nature of the study, the framework inevitably rests on simplifying assumptions that may introduce bias. These assumptions concern both the synthetic data generation process and the evaluation of machine learning models, and must be explicitly acknowledged.

At the data level, imbalance labels were derived from task-specific thresholds for dynamic H:Q ratio, knee moment LSI, and co-contraction index. Although grounded in sports medicine literature, such thresholds are debated and may over- or under-estimate imbalance prevalence. Similarly, the distribution of latent subject heterogeneity was modeled as bimodal Gaussian clusters, which simplifies real-world variation. While this provides a transparent dichotomy between balanced and imbalanced profiles, it may fail to capture intermediate or mixed phenotypes. In addition, ~5% random label noise was deliberately introduced to mimic misclassification. This design improves robustness but simultaneously injects uncertainty, potentially biasing the evaluation metrics. Certain aspects such as inter-muscular coordination complexity, stochastic measurement noise, and individual sensor-level variability could not be fully replicated in simulation but are recognized as areas for empirical validation.

At the modeling level, calibration and performance estimates relied on subject-wise cross-validation. Although this prevents trial leakage, it may still underestimate generalization error if real athletes exhibit greater biomechanical variability than the synthetic cohort. Bootstrap confidence intervals partially mitigate this, yet external validation on empirical datasets remains essential.

Finally, interpretability analyses (permutation importance, partial dependence) assume relative independence of features and monotonic relationships. While these visualizations enhance transparency, they may oversimplify complex interactions in real biomechanics.

Taken together, these sources of bias highlight that the present work should be viewed as a methodological demonstration, not a clinical validation. Explicit acknowledgment of these limitations strengthens reproducibility and clarifies the translational path toward application on real datasets. Taken together, these sources of bias highlight that the present work should be viewed as a methodological demonstration, not a clinical validation. Explicit acknowledgment of these limitations strengthens reproducibility and clarifies the translational path toward application on real datasets.

To provide a structured overview, the main sources of bias in the in silico framework are summarized in Table S8, together with their potential impact, severity, interpretability dimensions, evidence base, and mitigation strategies. This synthesis highlights where methodological simplifications may influence generalizability and how transparency, sensitivity analyses, and future validation can address these concerns.

Table S8. Potential sources of bias in the in silico framework, their impact, interpretability dimensions, and mitigation strategies.

Source of Bias / Assumption	Description	Potential Impact	Severity	Interpretability Dimension	Evidence Base	Mitigation Strategy
**Threshold-based labeling**	Fixed cut-offs for H:Q, LSI, CCI	May over- or under-estimate imbalance prevalence	Moderate	Labeling / class balance	Sports medicine debates on H:Q cut-offs and LSI ±10–15%	Sensitivity analysis across threshold ranges

**Synthetic subject distribution**	Bimodal Gaussian latent profiles	Simplifies real variability; may omit intermediate cases	High	Generalization / external validity	Common in simulation studies; lacks mixed phenotypes	Transparent reporting; future validation on real athletes
**Label noise (5%)**	Random flips introduced for robustness	Increases uncertainty in performance metrics	Low	Calibration / stability	Standard ML stress-test technique	Bootstrap CIs; robustness analysis with 0–10% noise
**Cross-validation scheme**	Subject-wise GroupKFold	May underestimate generalization error vs. real data	Moderate	Generalization	Recommended in biomechanics ML, but limited to synthetic cohorts	Strict grouping; external validation recommended
**Interpretability methods**	Permutation importance, partial dependence	May oversimplify feature interactions	Moderate	Feature transparency	Widely used in interpretable ML; known limitations	Use multiple methods; report global + local perspectives

Notes. Identified biases were systematically analyzed and reported to enhance transparency. Their explicit acknowledgment ensures reproducibility and provides a clear roadmap for future validation on empirical datasets.

Overall, the identified biases reflect the tension between methodological control and muscular complexity. By reducing imbalance to fixed cut-offs, approximating subject heterogeneity with synthetic distributions, and modeling co-contraction in simplified form, the framework inevitably abstracts away from the full richness of hamstring–quadriceps physiology. Yet, these simplifications are not arbitrary: they retain explicit links to clinical practice, where H:Q ratios, limb symmetry indices, and co-contraction thresholds continue to be debated and applied as return-to-sport criteria. Thus, the in silico approach provides a transparent environment to expose how muscular imbalance can be operationalized, evaluated, and interpreted. Rather than being viewed as limitations alone, these biases highlight the reproducibility of the methodology and its alignment with muscle-relevant constructs, while underscoring the need for empirical validation against real-world hamstring–quadriceps data.

Potential circularity—Labels were defined using thresholds on dynamic H:Q, knee moment LSI, and CCI. The same variables also available to the model as predictors. This overlap creates an inherent risk of circularity. We mitigated it by (i) explicitly reframing the contribution as calibration and continuous ranking of a clinically motivated composite rule, (ii) conducting no-leak ablations that excluded label-defining features, and (iii) benchmarking against a calibrated rule score. These steps ensure that the model's added value can be distinguished from the deterministic rule, even though future validation with latent targets independent of these features remains necessary.

S1.3.5 Anti-leak ablations and calibrated-rule comparator

To address the circularity arising from labels defined by dynamic H:Q, knee moment LSI, and CCI, we conducted subject-wise no-leak ablations. Four configurations were trained under identical GroupKFold (k=5), isotonic calibration, and cluster bootstrap (2,000×, resampling subjects):

- Full-features ML (baseline).
- No-label-features ML: all predictors except H:Q, LSI, and CCI.
- Label-features-only ML: using only H:Q, LSI, and CCI.
- Calibrated rule score: distance-to-threshold rule, isotonic-calibrated.

Metrics included ROC-AUC, PR-AUC, balanced accuracy, F1, Brier score, calibration slope/intercept, and net benefit (DCA). This quantified how much signal persists without label-defining variables and whether ML provides incremental value beyond a calibrated deterministic rule.

S1.4 Interpretability and Reporting

Permutation Importance—At the global level, permutation feature importance was applied to quantify the relative contribution of each biomechanical variable to model predictions. The method operates by randomly shuffling the values of a given predictor across the dataset, thereby breaking its association with the outcome, and measuring the resulting drop in classification performance. Unlike impurity-based importance scores derived from decision trees, this approach provides a model-agnostic estimate that more faithfully reflects predictive reliance under realistic perturbations.

The analysis consistently highlighted the hamstrings–quadriceps ratio ($H{:}Q_{dyn}$), limb symmetry index (LSI), and co-contraction index (CCI) as the dominant contributors to classification. This outcome was not only statistically robust but biomechanically meaningful: $H{:}Q_{dyn}$ values outside the physiological range (<0.6 or >1.2) are recognized indicators of muscular imbalance; LSI values exceeding 10–15% are clinically adopted thresholds in return-to-sport decision-making; and elevated CCI reflects compensatory neuromuscular control strategies. The emergence of these features as top-ranked determinants confirms that the classifier aligned with established biomechanical constructs rather than spurious artifacts of data generation.

By ranking predictors in physiologically interpretable order, permutation importance bridged the gap between abstract machine learning processes and biomechanical reasoning. For clinicians, it reassures that the model's predictions rely on the same constructs they evaluate in practice. For researchers, it provides evidence that the synthetic framework embedded clinical priors correctly and that predictive validity arose from genuine musculoskeletal asymmetries rather than noise.

Partial Dependence Plots—Partial dependence plots (PDPs) were employed to visualize the marginal effect of individual features on predicted imbalance risk while averaging over all other variables. By systematically varying the value of a single predictor and holding the remaining predictors constant, PDPs provide an interpretable mapping between feature values and model outputs. This technique exposes non-linearities and threshold behaviors that are not evident from global performance metrics alone.

The PDPs revealed clinically relevant patterns, such as a steep increase in predicted imbalance probability once the limb symmetry index (LSI) exceeded ~15%, consistent with thresholds commonly adopted in return-to-sport testing. Similarly, extreme deviations in dynamic hamstrings–quadriceps ratio ($H{:}Q_{dyn} < 0.6$ or > 1.2) were associated with disproportionately elevated risk, while moderate co-contraction index (CCI) values appeared protective, but excessive co-activation suggested compensatory strategies. These non-linear responses mirrored established biomechanical observations, underscoring that the model had internalized physiologically grounded decision boundaries.

By surfacing threshold effects directly within the probabilistic predictions, PDPs demonstrated that the classifier did not behave as a black box but instead recovered relationships long recognized in sports medicine. For practitioners, this means that model outputs can be linked to familiar clinical benchmarks, facilitating trust and adoption. For researchers, the results validate that the synthetic dataset embedded realistic biomechanical

structure, and that the machine learning pipeline preserved this structure rather than obscuring it.

Confusion Matrix—Confusion matrices were generated to provide a direct visualization of classification outcomes at the trial level. These matrices summarize predictions into true positives, true negatives, false positives, and false negatives, thereby capturing not only overall accuracy but also the distribution of specific error types. Unlike aggregate metrics such as ROC-AUC or F1-score, confusion matrices allow inspection of model behavior in concrete terms that are more easily interpretable by clinicians and applied scientists.

Inspection of the confusion matrices revealed that correctly classified imbalanced cases were predominantly those with pronounced deviations in $H:Q_{dyn}$ or LSI, aligning with clinically unambiguous asymmetries. Conversely, false negatives were more likely to occur in borderline athletes with mild asymmetry, a scenario that also poses diagnostic uncertainty in empirical sports medicine. False positives tended to arise in athletes whose stride-to-stride variability or co-contraction profiles mimicked imbalance under noisy conditions, highlighting that model misclassifications were not arbitrary but reflected the gray zones of clinical evaluation.

By exposing both the strengths and the weaknesses of the classifier, confusion matrices underscored the framework's transparency. For practitioners, they provided a tangible sense of how often an athlete might be misclassified and under what conditions. For researchers, they offered a diagnostic tool to identify systematic patterns in errors and opportunities for refinement. Together, confusion matrices complemented global metrics by translating predictive performance into biomechanically and clinically meaningful outcomes.

Error analysis—Beyond aggregate confusion matrices, error analysis was performed to characterize the nature of misclassifications in greater detail. Rather than treating false positives and false negatives as symmetric or interchangeable, we examined their distribution relative to class prevalence, feature thresholds, and trial-level variability. This approach enabled identification of systematic error patterns, which are often more informative for clinical translation than overall accuracy alone.

The analysis revealed that false negatives clustered among athletes with borderline values of $H:Q_{dyn}$ (≈0.55–0.65) or LSI (≈12–15%), reflecting the inherent ambiguity in defining strict cut-offs for imbalance. These "near-threshold" profiles are challenging even in clinical return-to-sport testing, where disagreement between assessors is common. False positives, on the other hand, frequently corresponded to trials with elevated stride-to-stride variability or atypical co-contraction patterns, which may mimic imbalance despite overall symmetrical strength. Such errors suggested that the classifier was sensitive to biomechanical noise sources that are likewise encountered in experimental gait analysis.

By contextualizing misclassifications in biomechanical terms, error analysis demonstrated that the model's errors were not random, but reflected the gray zones of clinical practice. For practitioners, this highlighted that borderline athletes remain difficult to classify reliably—whether by human experts or machine learning. For researchers, error patterns pointed to specific areas where additional features, improved noise modeling, or empirical data could strengthen the framework. Thus, error analysis transformed apparent

weaknesses into insights about both model limitations and real-world diagnostic uncertainty.

Probability Calibration—To ensure that predicted probabilities could be interpreted as clinically meaningful risks rather than arbitrary classifier scores, probability calibration was performed. Isotonic regression was applied to out-of-fold predictions, aligning estimated probabilities with observed event frequencies without assuming linearity in the log-odds space. Calibration plots and metrics such as slope, intercept, and Brier score were examined to verify that predicted likelihoods tracked empirical outcomes across the full probability spectrum.

Well-calibrated predictions ensured that, for example, an athlete assigned a 0.80 probability of imbalance indeed corresponded to a roughly 80% empirical chance of exceeding biomechanical thresholds such as $H{:}Q_{dyn} < 0.6$ or $LSI > 15\%$. Without calibration, such probabilities might systematically overestimate or underestimate actual risk, potentially misleading clinical decision-making. By demonstrating that predicted risks faithfully mapped onto threshold-based imbalance definitions, calibration confirmed that the classifier's outputs were not abstract numerical indices but reflected physiologically grounded likelihoods.

Calibration elevated the framework from a statistical classifier to a clinically interpretable decision-support tool. For practitioners, it meant that model outputs could be read as direct estimates of imbalance risk, facilitating transparent communication with athletes and rehabilitation teams. For researchers, it highlighted the importance of aligning machine learning predictions with empirical prevalence, ensuring that reported probabilities carry real-world meaning. In doing so, calibration reinforced the translational potential of the framework by bridging probabilistic modeling and biomechanical interpretation.

Reproducibility (code and data)—To safeguard transparency and enable independent verification, the entire pipeline was implemented as a modular and reproducible computational environment. All components of the framework—including the synthetic data generator, feature extraction routines, machine learning configuration, and interpretability analyses—were specified in code with fixed random seeds and version-controlled libraries. The synthetic dataset, together with configuration files and scripts, was archived to allow identical reruns of all experiments.

Reproducibility in this context extends beyond computational rigor to biomechanical credibility. By providing a synthetic cohort with explicit definitions of imbalance thresholds ($H{:}Q_{dyn}$, LSI, CCI), the framework ensured that future researchers could validate, challenge, or extend its assumptions under transparent conditions. The availability of code and data transforms the framework from a one-off proof-of-concept into a reusable methodological scaffold, preserving the biomechanical constructs that anchor its design.

For practitioners, reproducibility safeguards mean that findings are not idiosyncratic artifacts of an opaque algorithm, but can be consistently reproduced and scrutinized. For researchers, it enables comparative studies, benchmarking, and incremental refinement. This openness strengthens the credibility of in silico biomechanics and accelerates translation into empirical and clinical settings.

In summary, the interpretability analyses and reproducibility safeguards elevated the framework beyond predictive performance alone, ensuring that every output could be traced to biomechanical constructs, scrutinized through transparent error diagnostics, and replicated under controlled conditions. By integrating feature importance, partial dependence, confusion matrices, error analysis, probability calibration, and open reproducibility, the methodology provided not only a proof-of-concept classifier but a transparent and physiologically meaningful tool for studying hamstrings–quadriceps imbalance.
